# The RNA Chaperone Protein Hfq Regulates the Characteristic Sporulation and Insecticidal Activity of *Bacillus thuringiensis*

**DOI:** 10.3389/fmicb.2022.884528

**Published:** 2022-04-11

**Authors:** Zhaoqing Yu, Yang Fu, Wei Zhang, Li Zhu, Wen Yin, Shan-Ho Chou, Jin He

**Affiliations:** ^1^State Key Laboratory of Agricultural Microbiology, College of Life Science and Technology, Huazhong Agricultural University, Wuhan, China; ^2^National Engineering Research Center of Edible Fungi, Institute of Edible Fungi, Shanghai Academy of Agricultural Sciences, Shanghai, China

**Keywords:** *Bacillus thuringiensis*, Hfq, transcriptomics, motility, biofilm formation, sporulation, insecticidal activity

## Abstract

*Bacillus thuringiensis* (Bt) is one of the most widely used bio-insecticides at present. It can produce many virulence factors and insecticidal crystal proteins during growth and sporulation. Hfq, on the other hand, is a bacterial RNA chaperone that can regulate the function of different kinds of RNAs, thereby affecting various bacterial phenotypes. To further explore the physiological functions of Hfq in Bt, we took BMB171 as the starting strain, knocked out one, two, or three *hfq* genes in its genome in different combinations, and compared the phenotypic differences between the deletion mutant strains and the starting strain. We did observe significant changes in several phenotypes, including motility, biofilm formation, sporulation, and insecticidal activity against cotton bollworm, among others. Afterward, we found through transcriptome studies that when all *hfq* genes were deleted, 32.5% of the genes in Bt were differentially transcribed, with particular changes in the sporulation-related and virulence-related genes. The above data demonstrated that Hfq plays a pivotal role in Bt and can regulate its various physiological functions. Our study on the regulatory mechanism of Hfq in Bt, especially the mining of the regulatory network of its sporulation and insecticidal activity, could lay a theoretical foundation for the better utilization of Bt as an effective insecticide.

## Introduction

Hfq is a ubiquitous RNA chaperone protein in bacteria. It was first discovered as an essential host factor for Qβ phage RNA replication in *Escherichia coli*. It shares a high degree of structural similarity with the eukaryotic spliceosome Sm protein involved in RNA degradation (Franze de Fernandez et al., 1968; Valentin-Hansen et al., 2004; Sauer, 2013), and its monomeric secondary structure is relatively conserved in different bacteria, comprising a solid N-terminal Sm domain and a looser and more variable C-terminus (Schumacher et al., 2002), where, the Sm domain is the core region of Hfq for RNA binding, which is consisted of a Sm1 motif with β1-β3 strands and a Sm2 motif with β4-β5 strands (Beich-Frandsen et al., 2011; Vincent et al., 2012). Bacterial intracellular Hfq proteins all form cyclic hexameric structures, showing two asymmetric polar faces, namely the proximal and distal polar faces. The proximal face is the side with an exposed N-terminal (α-helix that preferentially binds small RNAs (sRNAs) carrying U-rich internal motifs and a U-tailed terminator, whereas the distal face is located on the other side of the proximal face and preferentially binds target mRNAs with A-rich motifs that are often present in the 5′-untranslated regions and ribosome-binding sites. With these two polar faces and rim face, Hfq is found to regulate various physiological activities of bacteria by binding to different kinds of RNAs (mainly sRNAs and mRNAs) and affects their stability or translation processes (Updegrove et al., 2010; Vogel and Luisi, 2011; De Lay et al., 2013; Djapgne and Oglesby, 2021). For example, Hfq is able to interact directly with some sRNAs (Møller et al., 2002), such as RyhB to increase its stability in regulating iron metabolism in *E. coli* (Massé et al., 2003), or RsmY to control the quorum sensing of *Pseudomonas aeruginosa* (Sonnleitner et al., 2006). Meanwhile, Hfq can also directly bind to certain mRNAs to control their translation, and ultimately affect the expression of specific proteins. For example, Hfq can affect bacterial intracellular c-di-GMP concentration and regulate bacterial biofilm formation through post-transcriptional repression of mRNA translation of the diguanylate cyclase-encoding gene *hms*T (Ellis et al., 2015; Chen and Gottesman, 2017; Fu et al., 2021). In addition, Hfq can also promote the base pairing of sRNA with its target mRNA, thereby regulating the expression level of target genes (Pfeiffer et al., 2009; Hämmerle et al., 2012), affecting bacterial growth and environmental adaptability (Vogt and Raivio, 2014), abrogating carbon catabolite repression (Sonnleitner and Bläsi, 2014), and altering bacterial virulence to the host (Dos Santos et al., 2019). Taken together, the diversity of Hfq regulatory mechanisms suggests that Hfq is indeed a flexible RNA mediator that can compete with hundreds of sRNA molecules and thousands of mRNA sequences for binding to affect various bacterial physiological activities mediated by these RNAs. Therefore, Hfq is a *bona fide* global post-transcriptional regulator in bacteria (Zhang et al., 2003; Fu et al., 2021).

*Bacillus cereus* (Bc) group is a group of *Bacillus* genus composed of closely related species, including *Bacillus anthracis, Bacillus cereus, Bacillus thuringiensis* (Bt), *Bacillus toyonensis*, etc (Liu et al., 2017). Among them, Bt is one of the most widely used bio-insecticides. It exhibits important difference from other bacterial species in that it can produce insecticidal crystal proteins (ICPs) during sporulation, thereby conferring its highly effective insecticidal activity against different insects (He et al., 2010; Bravo et al., 2011; Sanahuja et al., 2011). In addition to ICPs, Bt also contains other virulence factors commonly found in Bc group members, including lecithinase, enterotoxin, hemolysin, etc (Raymond et al., 2010; Liu et al., 2014, 2021). It is worth mentioning that the spores produced by Bt also exhibit certain synergistic effects on the insecticidal activity against target insects (Wang et al., 2013b). In the process of sporulation, many key regulatory genes are involved, such as *spo0A*, *sigH*, *sigF*, *sigE*, etc (Kroos et al., 1999), which can carry out temporal and spatial control in different sporulation phases in an orderly and accurate manner, and in coordination with many downstream sporulation-related genes (de Hoon et al., 2010; Zheng et al., 2015).

In the present study, we found the presence of three Hfq-encoding genes in the Bt BMB171 genome. We carried out combinatorial knockouts of different *hfq* genes using the Markerless gene knockout approach, including constructing a mutant without any *hfq* gene, and compared the phenotypic changes of these mutants to analyze the regulatory functions of Hfq in Bt. We also analyzed the effect of *hfq* deletion on the global transcript level by transcriptome sequencing (RNA-seq), mined target mRNAs downstream of Hfq, and explored the effect of Hfq on bacterial motility, biofilm formation, sporulation, and its insecticidal activity, hoping to lay a theoretical foundation for better utilization of Bt.

## Materials and Methods

### Strains, Plasmids, Primers, and Culture Conditions

Strains and related plasmids used in this study are listed in Tables 1, 2, respectively. We routinely cultured *Escherichia coli* in LB medium at 37°C and Bt in LB medium at 28°C and examined their growth curves. The final concentrations of antibiotics added to the medium used in this study were: ampicillin, 100 μg/mL; kanamycin, 50 μg/mL; spectinomycin at 300 μg/mL and 100 μg/mL for Bt and *E. coli*, respectively. Restriction endonucleases were purchased from Takara Company, and DNA polymerases from CWbio Company. The PCR-related primers involved in this study are listed in Supplementary Table 1. All PCR experiments were carried out using the genomic DNA of the Bt starting strain BMB171 (GenBank accession number NC_014171NC_014171) or BMB171-derived strains as templates (He et al., 2010).

**TABLE 1:** Strains used in this study.

Strains	Description	References
*E. coli* DH5α	A cloning host	Beijing TransGen Biotech Co., Ltd.
DH5α/pSS1827	DH5α with pSS1827, used for knocking out of gene in BMB171	Janes and Stibitz, 2006
DH5α/pSS4332	DH5α with pSS4332, used for knocking out of gene in BMB171	Janes and Stibitz, 2006
DH5α/pRP1028	DH5α with pRP1028, used for knocking out of gene in BMB171	Janes and Stibitz, 2006
DH5α/pRP1028-*hfq1*UD	DH5α with pRP1028-*hfq1*UD, used for knocking out of *hfq1* in BMB171	This work
DH5α/pRP1028-*hfq2*UD	DH5α with pRP1028-*hfq2*UD, used for knocking out of *hfq2* in BMB171	This work
DH5α/pRP1028-*hfq3*UD	DH5α with pRP1028-*hfq3*UD, used for knocking out of *hfq3* in BMB171	This work
BMB171	An acrystalliferous mutant strain of Bt with high transformation frequency (NC_014171)	He et al., 2010
Δ*hfq1*	*hfq1* mutant of BMB171	This work
Δ*hfq2* (Δ1*hfq*)	*hfq2* mutant of BMB171	This work
Δ*hfq3*	*hfq3* mutant of BMB171	This work
Δ*hfq1*Δ*hfq2* (Δ2*hfq*)	*hfq1* and *hfq2* double mutant of BMB171	This work
Δ*hfq2*Δ*hfq3*	*hfq2* and *hfq3* double mutant of BMB171	This work
Δ*hfq1*Δ*hfq3*	*hfq1* and *hfq3* double mutant of BMB171	This work
Δ*hfq1*Δ*hfq2*Δ*hfq3* (Δ3*hfq*)	*hfq1*, *hfq2*, and *hfq3* triple mutant of BMB171	This work
BMB171+	BMB171 with pHT1K*-*Flag	This work
BMB171+*katB*	BMB171 with pHT1K- P*katB-katB-*Flag	This work
Δ1*hfq*+*hfq2*	Δ1*hfq* with pHT1K- P*hfq-hfq2-*Flag	This work
Δ2*hfq*+*hfq2*	Δ2*hfq* with pHT1K- P*hfq-hfq2-*Flag	This work
Δ3*hfq*+*hfq2*	Δ3*hfq* with pHT1K-P*hfq-hfq2-*Flag	This work
BMB171-*cry*	BMB171 containing plasmid pBMB43-304, which expressed Cry1Ac10 in BMB171	This work
Δ3*hfq-cry*	Δ3*hfq* containing plasmid pBMB43-304, which expressed Cry1Ac10 in Δ3*hfq*	This work

**TABLE 2:** Plasmids used in this study.

Plasmids	Description	References
pSS1827	the helper plasmid for transconjugation, *amp^r^*	Janes and Stibitz, 2006
pSS4332	*Bacillus-E. coli* shuttle plasmid; containing *gfp* I-SceI restriction enzyme encoding genes, *kan^r^*	Janes and Stibitz, 2006
pRP1028	*Bacillus-E. coli* shuttle plasmid; containing *turbo-rfp* gene and an I-SceI recognition site, *amp*^r^*spc*^r^**	Janes and Stibitz, 2006
pRP1028-*hfq1*UD	pRP1028 with the upstream and downstream regions of *hfq1*, used for *hfq1* deletion	This work
pRP1028-*hfq2*UD	pRP1028 with the upstream and downstream regions of *hfq2*, used for *hfq2* deletion	This work
pRP1028-*hfq3*UD	pRP1028 with the upstream and downstream regions of *hfq3*, used for *hfq3* deletion	This work
pBMB43-304	pHT304 carrying *cry1Ac10*, used for expression of Cry1Ac10 protein in Bt	Qi et al., 2015
pHT1K-Flag	pHT1K vector harboring the Flag tag	Wang et al., 2013a
pHT1K-P*katB-katB-*Flag	*katB* with promoter in *Hin*dIII and *Pst*I sites of pHT1K-Flag	This work
pHT1K-P*hfq-hfq2-*Flag	*hfq2* with promoter in *Hin*dIII and *Pst*I sites of pHT1K-Flag	This work

### Markerless Gene Knockout Method to Construct Mutants

The primers for constructing the above-mentioned knockout mutants are listed in Supplementary Table 1; they were used to amplify the upstream and downstream sequences of the target genes to facilitate double crossover by homologous recombination. We also made appropriate improvements to the methods reported in the literature (Janes and Stibitz, 2006) to obtain a protocol suitable for knocking out genes in Bt (Zheng et al., 2015), and the target mutants were verified by PCR and sequencing (Fu et al., 2018).

### Bacterial Motility Assays

All tested strains were inoculated at the same initial concentration and grown under the same culture conditions. After shaking on a shaker for 9 h, 5 μL of the bacterial liquid culture in the mid-logarithmic phase was taken and dropped onto the center of the plate in semi-solid medium (LB containing 0.5% agar), followed by standing for 10–15 min. After the bacterial liquid became solidified, it was transferred to a 28°C incubator for further culturing for 8 h (Fu et al., 2018).

### Quantification Assays of Biofilm

The determination of biofilm amount was appropriately optimized with reference to the method used for *Clostridium difficile* (Bordeleau et al., 2011): the strains to be tested were cultured with shaking in glass bottles of LB liquid medium for 12 h, and then left to stand for another 36 h. After gently removing the bacterial liquid culture, the glass bottles were washed twice with ultrapure water. Adherent biofilm was then stained with 1% crystal violet for 30 min at room temperature for quantification. After washing off the residual dye with ultrapure water and allowing the glass bottles to dry, the crystal violet-stained biofilm was re-suspended in 2 ml of 96% (v/v) ethanol and the absorbance of purple suspension at 595 nm was measured (Tang et al., 2016).

### Spore Counting

The spore counting method was taken from that reported in the references (Wang et al., 2016; Li et al., 2020) and adjusted as follows: different strains were cultured in LB medium, and then 1 mL of each sample was taken out and placed in an 80°C water bath for 10 min. After appropriate serial dilutions, 100 μL of each diluent was spread onto LB plates to count the CFU (colony-forming units) per mL. The calculation formula of the spore numbers is as follows: the actual spore numbers = (the single colony numbers on the plate × the dilution factor × the initial volume of the bacterial suspension)/the spreading volume of the bacterial suspension.

### Scanning Electron Microscopy

The scanning electron microscopy imaging method in this study comes from the literature (Tang et al., 2016). Bt cultured in LB medium for 9 or 27 h were dropped on a microscope slide and placed at 28°C in an incubator for 27 h. The samples were then fixed with 4% glutaraldehyde for 2 h and dehydrated with ethanol solutions of different concentrations. The samples were finally freeze-dried and imaged using a JSM-6390 Scanning electron microscopy (JEOL, Japan).

### Bacterial Insecticidal Activity Assays

Tested strains BMB171, Δ3*hfq*, BMB171-*cry*, and Δ3*hfq-cry* were grown under the same conditions, and the bacterial cultures in the late stationary phase or early sporulation phase (48 h) were centrifuged at 10,000 × *g*, 4°C for 5 min for collection of pellets, which were re-suspend with an appropriate amount of ultrapure water to obtain properly diluted bacterial suspensions before adding it to the artificial feed. The artificial diet formula for breeding cotton bollworm (*Helicoverpa armigera*) is: 4 g of yeast extract, 7 g of soybean meal, 0.5 g of vitamin C, 1.5 g of agar, 1.5 mL of 36% acetic acid and 2 g of penicillin in 100 mL of artificial feed medium (Tang et al., 2016). The artificial feed was then dispensed into 24-well cell culture plates (Costar, United States) at 1 mL/well, and 100 μL/well of the bacterial suspension to be tested was added to the culture plate. Each group of 72 cotton bollworm larvae was then placed in 24-well culture plates, with the survival rate recorded daily. After 7 days of continuous observation, the body length and body weight of the larvae were measured.

### RT-qPCR Assays

First, total RNA was isolated from Bt cells grown in LB liquid medium to specific time points using TRIzol reagent (Life Technologies, United States). Transcription of the first cDNA was carried out using the PrimeScript RT kit with gDNA Eraser (Takara Biotechnology, Japan) and used as the DNA template for real-time quantitative PCR (RT-qPCR) as previously described (Zheng et al., 2020) using *gapdh* (encoding glyceraldehyde 3-phosphate dehydrogenase) gene as the reference (Løvdal and Saha, 2014). Finally, we used the comparative C_*t*_ method to calculate changes in gene expression.

### RNA-Sequencing and Transcriptome Data Analysis

BMB171 and Δ3*hfq* cultured for 48 h were selected as transcriptome sequencing samples, with two biological replicates set for each sample. Total RNA was extracted with TRIzol reagent (Life Technologies, United States), and after appropriate processing, paired-end sequencing was carried out using an Illumina HiSeq TM 2500 sequencer (Illumina, United States). Sequencing was obtained by strand-specific RNA-seq using the Illumina Genome Analyzer IIx sequencing platform (Li et al., 2017). The raw data were deposited in NCBI under the Sequence Read Archive (SRA) with the BioProject number PRJNA811318 and SRA accession number SRP361911. The raw data were collected and filtered by FASTX-Toolkit^1^ to get a clean data set that could be further analyzed and processed to clean-reads with an average length of 100 nt. Then, Bowtie2 (version 2.2.3) was used to map the clean-reads of each sample to the unigene of the reference genome with an e threshold set to 0.00001 using the “-N<*1*>” parameter (Langmead and Salzberg, 2012), allowing clean-reads to be mapped to the genome with only one mismatch and reads matched to rRNA were removed. The R language package was used to calculate the numbers of reads mapped to each gene (Mortazavi et al., 2008). At the same time, the numbers of reads were normalized to Reads Per Kilo bases per Million reads (RPKM), which are the main data for analyzing gene transcription level.

Statistical analysis was performed on the processed data, and differentially expressed genes (DEGs) were filtered out using the MA-plot-based method with Random Sampling model (MARS) approach in the DEGseq package (Wang et al., 2010). The *p*-value was obtained from the differential gene expression tests. Benjamini’s False Discovery Ratio (FDR) operation was used to determine *p*-value thresholds in multiple tests and analyses (Benjamini and Hochberg, 1995). Both FDR ≤ 0.001 and the absolute value of a ≥2-fold change [|log2(RPKM-Δ*3hfq*/RPKM-BMB171) normalized| ≥ 1] were used as thresholds to identify significant DEGs. The R language package was used to draw a volcano plot for the overall distribution of DEGs between the two samples.

## Results

### Distribution of *hfq* Genes in *Bacillus thuringiensis*

We have analyzed the distribution of Hfq in the genus *Bacillus* and found that Hfq is widely distributed, present in 78.5% of the species in the genus with fully sequenced genomes (Supplementary Table 2). Through Pfam (Mistry et al., 2021) and NCBI’s Conserved Domain Database (CDD) (Yang et al., 2020), we analyzed the copy numbers of *hfq* genes in some strains of Bc group with fully sequenced genomes and found that *hfq* is widely distributed in the Bc group, and the copy numbers of *hfq* genes are greater than 2 (Supplementary Table 3) with relatively conserved upstream and downstream genes (Vilas-Boas et al., 2007). We defined the Hfq present in most bacteria as the main Hfq, while the other Hfq that also occurs recurrently but less frequently as the secondary Hfq. Intriguingly, the upstream gene of the main Hfq coding gene is usually the coding gene of a hypothetical protein of unknown function, while the downstream gene is usually *mia*A (encoding tRNA Δ^2^-isopentenylpyrophosphate transferase) (Vrentas et al., 2015). In contrast, the upstream and downstream genes of secondary *hfq* typically encode sporulation regulatory protein and HAD superfamily hydrolase, respectively (Supplementary Figure 1).

**FIGURE 1:**
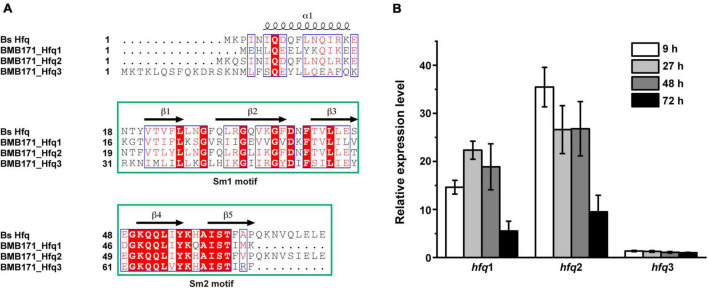
Sequence alignment and gene expression of three Hfq proteins in BMB171. **(A)** Secondary structure analysis of the three Hfq proteins in BMB171. Their amino acid sequences all contain two motifs, with Sm1 containing the conserved β1-β3 strands and Sm2 containing the β4-β5 strands. **(B)** Gene expression levels of the three *hfq* genes in BMB171 at different growth phases. Samples were taken at 9 h (logarithmic phase), 27 h (early stationary phase), 48 h (late stationary phase or early sporulation phase), and 72 h (final sporulation phase).

The starting strain of this study, Bt BMB171, has three copies of *hfq* gene: *BMB171_RS08230* and *BMB171_RS18400* are located on the chromosome, while *BMB171_RS27540* is located on the only large plasmid pBMB171 (He et al., 2010). We then defined them as *hfq*1, *hfq*2, and *hfq*3 according to their locus numbers, in which, *hfq*2 is the main *hfq* gene and *hfq*1 is the secondary *hfq* gene. By comparing the amino acid sequences of these three Hfq proteins with that of *Bacillus subtilis* (Bs) (Someya et al., 2012), we found that their secondary structures all exhibited high similarity and contained two highly conserved core functional motifs, Sm1 and Sm2 (Figure 1A). Next, the transcript levels of the three *hfq* genes were measured by RT-qPCR at different growth phases, such as at 9 h (logarithmic growth phase), 27 h (early stationary phase), 48 h (late stationary phase or early sporulation phase), and 72 h (final sporulation phase) (Supplementary Figure 2), which showed that *hfq*1 and *hfq*2 genes located on the chromosome were well expressed in all four growth phases. Among them, the expression level of the main gene *hfq*2 was higher than that of *hfq*1, and the *hfq*2 transcript was the highest in the logarithmic phase. However, transcription of *hfq*3 on plasmid pBMB171 was very low in all four growth periods (Figure 1B).

### Hfq Regulates the Motility of *Bacillus thuringiensis*

**FIGURE 2:**
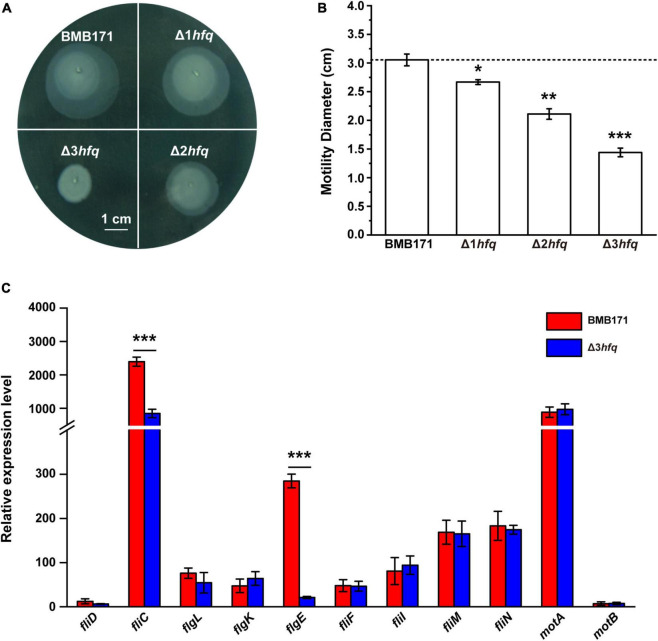
Deletion of *hfq* results in reduced Bt motility. **(A)** Motility assay of BMB171, Δ1*hfq*, Δ2*hfq*, and Δ3*hfq* on LB plates containing 0.5% agar. The scale bar is 1 cm. **(B)** Colony diameters of different strains. Motilities of BMB171, Δ1*hfq*, Δ2*hfq*, and Δ3*hfq* were correspondent to colony diameter. **(C)** RT-qPCR measurements of 11 flagella formation-related genes in BMB171 and Δ3*hfq*. The values are means ± SDs for triplicate assays. Significances of differences by Student’s *t*-test are indicated. ***p < 0.001; **p < 0.01; *p < 0.05.

To investigate the regulatory function of Hfq in Bt, we first constructed a series of *hfq* deletion mutants in combination in BMB171 using a homing endonuclease I-SceI-mediated Markerless gene knockout method, including three *hfq* single gene mutants (Δ*hfq*1, Δ*hfq*2, and Δ*hfq*3), three *hfq* double knockout mutants (Δ*hfq*1Δ*hfq*2, Δ*hfq*1Δ*hfq*3, and Δ*hfq*2Δ*hfq*3), and one *hfq* triple knockout mutant (Δ*hfq*1Δ*hfq*2Δ*hfq*3). We carried out various experiments to determine the phenotypes of these seven mutants, such as their motility (Supplementary Figure 3) and biofilm formation (Supplementary Figure 4), and obtained three mutants with a clear “cumulative effect” in phenotype, named Δ1*hfq* (Δ*hfq*2), Δ2*hfq* (Δ*hfq*1Δ*hfq*2), and Δ3*hfq* (Δ*hfq*1Δ*hfq*2Δ*hfq*3). We then carried out the corresponding motility assays for BMB171 and its mutants Δ1*hfq*, Δ2*hfq*, and Δ3*hfq*. As can be seen in Figure 2A, the motilities of the Δ1*hfq*, Δ2*hfq*, and Δ3*hfq* strains were gradually decreased as the deletion copy numbers of *hfq* increased (Figure 2B). Since bacterial motility is primarily regulated by flagella, the expression levels of genes involved in flagellar synthesis must be critical for bacterial motility (Mukherjee and Kearns, 2014; Ward et al., 2019). To this end, we measured the transcript levels of flagella-related genes (Supplementary Figure 5) in BMB171 and Δ3*hfq* and found that the expression levels of *fliC* and *flgE* associated with flagellar hooks and flagellar filaments were significantly reduced in Δ3*hfq* (Figure 2C). Therefore, it is thought that in Bt, Hfq may affect bacterial motility by regulating the transcription and expression of flagella-related genes.

### Hfq Affects *Bacillus thuringiensis* Biofilm Formation

**FIGURE 3:**
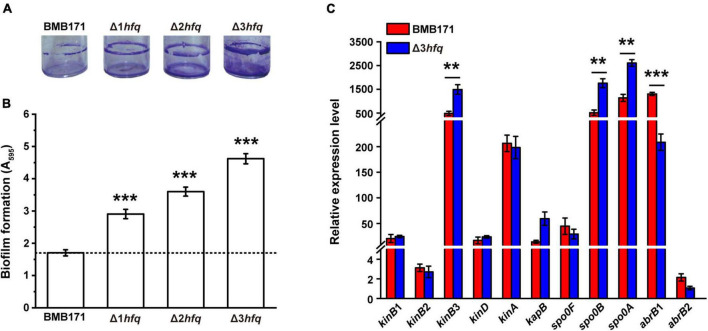
Deletion of *hfq* results in increased biofilm formation. **(A)** Representative photographs of biofilm formation assays for the BMB171, Δ1*hfq*, Δ2*hfq*, and Δ3*hfq* strains in glass bottles. **(B)** Quantification of biofilm formation for BMB171, Δ1*hfq*, Δ2*hfq*, and Δ3*hfq* by crystal violet stain measured by UV spectrophotometer at 595 nm. **(C)** RT-qPCR measurements of 11 genes associated with biofilm formation in BMB171 and Δ3*hfq*. The values are the mean ± SD for triplicate assays. Significances of differences by Student’s *t*-test are indicated. ***p < 0.001; **p < 0.01; *p < 0.05.

When Bt is applied as an insecticide, it must stay on the plant surfaces to produce insecticidal activity. Improving the ability of bacterial biofilm formation will thus enhance their adhesion to the plant surfaces and ensure the insecticidal performance of Bt (Jha et al., 2021). Therefore, we determined the amount of biofilm formation in Δ1*hfq*, Δ2*hfq*, and Δ3*hfq*. As can be seen in Figure 3A, from BMB171, Δ1*hfq*, Δ2*hfq*, to Δ3*hfq*, the amount of biofilm formation showed a gradient rise with the increase of *hfq* deletion copy, and the quantitative experimental results also confirmed that this phenotype exhibited a “cumulative effect” (Figure 3B). We also quantified the transcript levels of genes involved in biofilm formation (Supplementary Figure 6) in BMB171 and Δ3*hfq* using RT-qPCR (Figure 3C), and found that the transcript levels of *kinB*3, *spo0B*, and *spo0A* genes that promote biofilm formation were significantly increased. On the other hand, the transcript level of *abrB*1, encoding a transcription factor that has been shown to inhibit Bs biofilm formation (Weng et al., 2013; Mielich-Süss and Lopez, 2015), was significantly decreased, which resulted in a significant increase in the amount of biofilm of Δ3*hfq*. We therefore believe that Hfq plays an important role in regulating the biofilm formation of Bt.

### Hfq Affects *Bacillus thuringiensis* Sporulation

**FIGURE 4:**
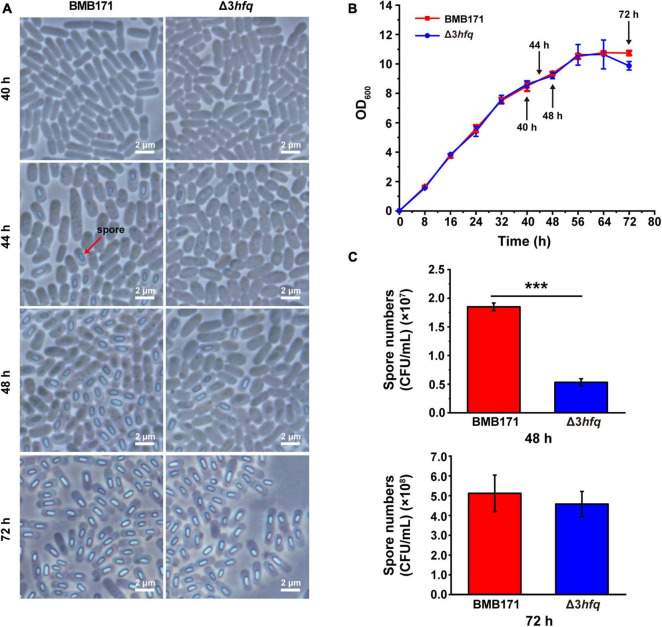
Deletion of *hfq* results in delayed sporulation. **(A)** Morphologies of spores in BMB171 and Δ3*hfq* was visualized by phase-contrast microscope at 40, 44, 48, and 72 h. The scale bar is 2 μm. **(B)** Growth curves of BMB171 and Δ3*hfq*. **(C)** Spore counting results of BMB171 and Δ3*hfq* at 48 and 72 h. The values are means ± SDs for triplicate assays. The significances of differences by Student’s *t*-test are indicated. ***p < 0.001; **p < 0.01; *p < 0.05.

We have used phase-contrast microscope (Figure 4A) and scanning electron microscope (Supplementary Figure 7) to observe the cell morphology of BMB171 and Δ3*hfq* strains, respectively, and found that the deletion of *hfq* did not affect the morphology of bacteria. Subsequently, we determined the growth curves of BMB171 and Δ3*hfq* in LB medium (Figure 4B), and there seems to be no significant difference in the growth rate and growth cycle between the two neither. In nutrient-rich LB medium, the entire growth cycle of BMB171 was approximately 72 h, and the time point to enter the late stationary phase or early sporulation phase was approximately 48 h. We observed sporulation of BMB171 and Δ3*hfq* cultured for 40, 44, 48, and 72 h, respectively, and found: (a) At 40 h, neither BMB171 nor Δ3*hfq* had sporulation; (b) At 44 h, BMB171 started to sporulate, but Δ3*hfq* had no obvious sporulation; (c) At 48 h, the spore numbers formed in BMB171 were significantly more than that in Δ3*hfq*; (d) By 72 h, the spore numbers formed in BMB171 were similar to that in Δ3*hfq* (Figure 4A). We counted the spore numbers in BMB171 and Δ3*hfq* at 48 and 72 h, respectively. As can be seen that at 48 h, the spore numbers in BMB171 were nearly three times higher than that in Δ3*hfq* with a significant difference; however, by 72 h, they were not significantly different (Figure 4C). Combined with these experimental results, we believe that the deletion of *hfq* in Bt delays the sporulation, but does not affect the cell morphology or growth rate of Bt.

### Hfq Affects the Insecticidal Activity of *Bacillus thuringiensis*

To explore the effect of Hfq on the insecticidal activity of Bt, we carried out insecticidal activity assays using larvae of the cotton bollworm *Helicoverpa armigera* that belongs to the family Noctuidae, order Lepidoptera, and is widely distributed in cotton and vegetable growing regions around the world (Lone et al., 2018). The ICP formed by Bt is the main component of its insecticidal activity against Lepidoptera insects (Baum and Malvar, 1995; Wang et al., 2013c). Since the starting strain BMB171 in this study is a mutant that does not produce any ICPs (He et al., 2010), we transferred the ICP encoding gene *cry1Ac10* into BMB171 and Δ3*hfq*, respectively (Wu et al., 2000; Deng et al., 2014), to construct recombinant strains BMB171-*cry* and Δ3*hfq-cry* capable of producing ICP. Although both BMB171 and Δ3*hfq* do not produce ICPs, there are still some toxins, such as enterotoxins, hemolysins, phospholipases, and proteases that affect the normal growth of the host (Bravo et al., 2011). We fed cotton bollworm larvae with a diet containing suspensions of bacteria BMB171, Δ3*hfq*, BMB171-*cry* and Δ3*hfq-cry* individually and observed their larval survival rates, body weights and body lengths 7 days later.

**FIGURE 5:**
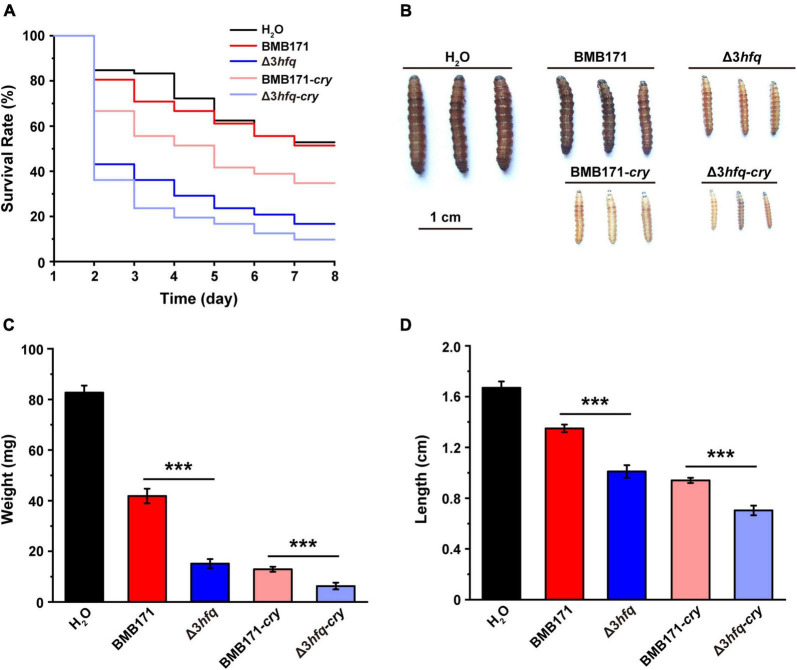
Deletion of *hfq* results in enhanced insecticidal activity. **(A)** Survival rates of cotton bollworm larvae fed with different strains for 7 days. **(B)** Morphological characteristics of cotton bollworm larvae after 7 days of feeding. **(C)** Weights of cotton bollworm larvae after 7 days of feeding. **(D)** Lengths of cotton bollworm larvae after 7 days of feeding. The values are means ± SDs for triplicate assays. Significances of differences by Student’s *t*-test are indicated. ***p < 0.001; **p < 0.01; *p < 0.05.

Compared with those fed with suspensions containing BMB171 and BMB171-*cry*, *H. armigera* larvae fed with Δ3*hfq* and Δ3*hfq-cry* suspensions resulted in significantly reduced survival rates (Figure 5A), decreased body weights (Figures 5B,C), shorter body length (Figures 5B,D), and substantially increased growth inhibition (Figure 5B). These results indicate that Hfq plays a rather important role in regulating the insecticidal activity of Bt. In addition, it can also be seen from Figure 5 that the inhibitory effect of strains BMB171-*cry* and Δ3*hfq-cry* on cotton bollworm larvae is even more obvious, which is mainly due to the expression of the Cry1Ac10 ICP.

### Effects of Hfq on the Transcriptional Regulation of *Bacillus thuringiensis*

As a bacterial RNA chaperone, Hfq may also play a key role in bacterial post-transcriptional regulation. To comprehensively investigate the global regulatory role of Hfq on Bt, we carried out RNA-seq on BMB171 and Δ3*hfq*. Since Δ3*hfq* has the characteristics of delayed sporulation and enhanced insecticidal activity compared with BMB171 when grown in LB medium for 48 h, samples at this time point were also chosen for RNA-seq to analyze the similarities and differences in post-transcriptional regulation of BMB171 and Δ3*hfq*. The raw transcriptome data were processed correspondingly and used to product a volcano plot (Supplementary Figure 8). Comparing the two obtained data sets, we found that the mutant Δ3*hfq* strain had a total of 1730 DEGs (32.5% of the total gene numbers), of which 1414 genes were significantly downregulated and 316 genes significantly upregulated.

**FIGURE 6:**
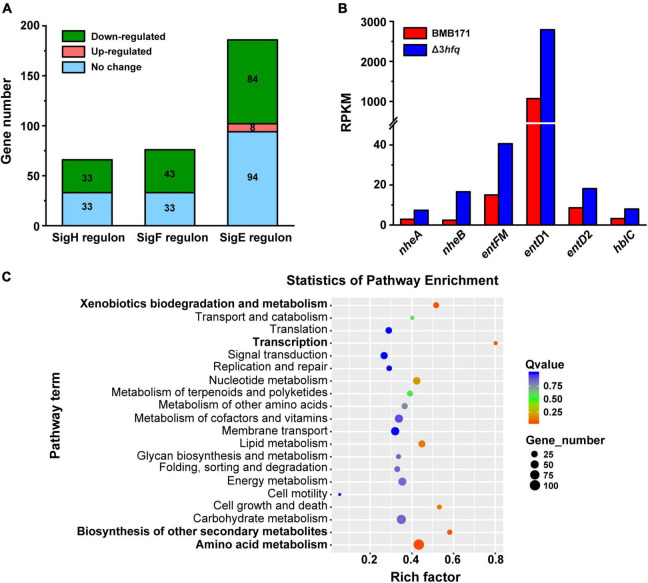
Transcriptome statistics of genes associated with sporulation affected by Hfq deletion. **(A)** Statistics of differential expression of downstream genes regulated by the sigma factors SigH, SigF, and SigE that control sporulation by BMB171 and Δ3*hfq*. **(B)** Difference in gene expression of related virulence factors in BMB171 and Δ3*hfq*. **(C)** Enrichment map of KEGG pathway of BMB171 and Δ3*hfq* DEGs. The names of the four significantly enriched pathways are shown in bold.

Starting from the transcriptome data, we first analyzed the transcription of sporulation-related genes in BMB171 and Δ3*hfq*. As can be seen from Figures 4A,C, deletion of Hfq delayed BMB171 sporulation, so we focused our analysis on the transcription of genes associated with early sporulation phase. At this stage, the sigma factors SigH, SigF, and SigE are sequentially activated (Kroos et al., 1999; Wang et al., 2013b) to regulate the transcription of other sporulation-related genes. Combined with DBTBS database^2^, we analyzed and identified related genes regulated by SigH, SigF, and SigE in BMB171. At the same time, we also combined transcriptome data to analyze the expression changes of these related genes at the transcriptional level (Supplementary Table 4) and found that: (a) Comparing the transcript levels of 66 genes regulated by SigH in BMB171, 33 genes were downregulated in Δ3*hfq*, while the transcript levels of the remaining genes exhibited no significant change; (b) 76 genes were regulated by SigF and located in the prospore, of which 43 genes were downregulated in Δ3*hfq*, and the other 33 genes exhibited no significant changes; (c) 186 genes located in the vegetative cells were regulated by SigE, and in Δ3*hfq*, 84 genes were downregulated, 94 remained unchanged, and 8 genes were upregulated (Figure 6A).

RNA-sequencing results showed that more than 50% of the sporulation-related genes in Δ3*hfq* were downregulated, which was consistent with the spore counting results of BMB171 and Δ3*hfq* (Figure 4C), further confirming that deletion of *hfq* gene directly caused delayed sporulation. These data indicate that Hfq is an important regulator of early sporulation phase.

In addition, the results of RNA-seq also showed that genes related to the synthesis of virulence factor enterotoxin, such as *nheA* (encoding non-hemolytic enterotoxin lytic component L2), *nheB* (encoding non-hemolytic enterotoxin lytic component L2) (Lindbäck et al., 2004), *entFM* (encoding enterotoxin), *entD*1, *entD*2 (encoding enterotoxin/cell-wall binding protein), and *hblC* (encoding hemolysin BL lytic component L2) (Ngamwongsatit et al., 2008) were all significantly upregulated in Δ3*hfq* with a log2 fold change of 1.59, 5.82, 1.71, 1.61, 1.1, and 1.5, respectively (Figure 6B). This result well explained that the insecticidal activity of Δ3*hfq* was higher than that of BMB171, and the insecticidal activity of Δ3*hfq-cry* was higher than BMB171-*cry* (Figure 5), and also confirmed that Hfq was an important regulator of bacterial virulence in the Bc group.

Other than validating and explaining the mechanism by which Hfq affects phenotypes such as sporulation and insecticidal activity, we also mined RNA-seq data through pathway enrichment analysis of KEGG and identified the main physiological metabolic pathways and signal transduction pathways involved in DEGs. We used the KEGG Orthology-Based Annotation System (KOBAS) 3.0 Program^3^ to enrich the known DEGs for the KEGG pathway, with the obtained results shown in the enrichment bubble analysis graph (Figure 6C). In this figure, four pathways, namely, the amino acid metabolism, xenobiotics biodegradation and metabolism, biosynthesis of other secondary metabolites, and transcription were significantly enriched. In addition, enriched pathways with *p*-values less than 0.05 included lipid metabolism and cell growth and death (Supplementary Table 5 lists all the DEGs enriched in these metabolic pathways). As can be seen from Supplementary Table 5, most of these DEGs were downregulated. Therefore, we hypothesized that Hfq inhibits these metabolic pathways. This result implied that Hfq is mainly involved in the regulation of amino acid metabolism, xenobiotics biodegradation and metabolism, biosynthesis of other secondary metabolites, and transcription.

## Discussion

### Significance of Multiple Copies of Genes Encoding Hfq in the *Bacillus cereus* Group

This study analyzed the copy numbers of *hfq* genes in Gram-positive Bc group bacteria (Supplementary Table 3), and found that they all contained two or more *hfq* genes, which is different from most Gram-negative bacteria such as *E. coli* (Mohanty et al., 2004) and *P. aeruginosa* (Sonnleitner et al., 2003) that contains only a single *hfq* gene. In Bt BMB171, there are a total of three *hfq* genes, and our results showed that the main gene is *hfq*2, and the deletion of which significantly affected bacterial motility (Supplementary Figure 3) and biofilm formation (Supplementary Figure 4). Furthermore, *hfq*1 played a relatively smaller role, and *hfq*3 was generally not transcribed (Figure 1B). When we complemented the *hfq*2 gene back to the Δ1*hfq*, Δ2*hfq*, and Δ3*hfq* mutants, respectively, we found that complementation of the *hfq*2 gene did not fully restore the reduced motility in Δ2*hfq* and Δ3*hfq* as revealed in the motility phenotype experiments, suggesting that *hfq*1 and *hfq*3 in BMB171 also exhibited some other functions (Supplementary Figure 9). We hypothesize from this data that the three *hfq* genes in BMB171 are all valid copies and can function physiologically under certain specific conditions, for example, in a relatively harsh living environment. If Bt lost its main *hfq* gene due to external stress, the remaining copies may function to survive external pressure to sustain themselves. This is not only related to the degree of bacterial evolution, but also reflects that Hfq is very important for the entire Bc group bacteria to respond to different external environments.

### Physiological Activity Regulation Network of Hfq on *Bacillus thuringiensis*

Our results demonstrated that Bt strains lacking *hfq* exhibited multiple phenotypic changes, including decreased motility, increased biofilm formation, delayed sporulation, and enhanced insecticidal activity. Through the KEGG Pathway enrichment analysis of DEGs, we found that in Bt, Hfq could participate in the regulation of multiple metabolic pathways, among which amino acid metabolism, xenobiotics biodegradation and metabolism, biosynthesis of other secondary metabolites, and transcription were significantly enriched (Figure 6C), indicating that Hfq can participate in the regulation of these four metabolic pathways in bacteria. In addition, two metabolic pathways, lipid metabolism and cell growth and apoptosis, may also be regulated by Hfq. The above results fully demonstrated that Hfq can play a pivotal role of comprehensively regulating the physiological activities of Bt. Hfq had also been shown to regulate bacterial motility (Schachterle et al., 2019), biofilm formation (Irie et al., 2020), and virulence in some Gram-negative bacteria (Pearl Mizrahi et al., 2021), suggesting that the regulation of certain physiological activities in bacteria by Hfq is universal. However, in Bs, deletion of Hfq was found to affect relatively fewer physiological functions, such as bacterial survival (Rochat et al., 2015), motility, chemotaxis (Jagtap et al., 2016) and cellulolysis etc. (Yang et al., 2021); besides, the deletion of Hfq exhibits no overall effect on the bacterial transcriptome, but only on certain distinct regulons, such as the ResD-ResE signal transduction system required for aerobic and anaerobic respiration, the GerE regulon encoding late spore coat genes and the ComK regulon regulating competence and DNA uptake (Hämmerle et al., 2014). These results suggested that some functions of Hfq are specific and Hfq may regulate different physiological activities in different bacteria even in the same genus.

The above discussion shows that Hfq is universal and specific in regulating bacterial physiological activities. Although we already have a rich understanding about the function of Hfq serving as an RNA chaperone protein, how Hfq regulates these physiological activities by interacting with RNA molecules remains unknown, that is, the molecular mechanism of how Hfq exerts its regulatory role has not yet been fully elucidated. Therefore, we aim to build a complete Hfq regulatory network in Bt in future studies.

### The Effect of Hfq on the Insecticidal Activity of *Bacillus thuringiensis* Will Promote the Development of Its Application

This study showed that the insecticidal activity of Δ3*hfq* against cotton bollworm was significantly enhanced than that of BMB171, indicating that Hfq played a regulatory role on the insecticidal activity of Bt. For many bacterial pathogens, virulence is a very complex and integrative phenotype. In other words, the virulence system is the result of the joint action of many related phenotypes (Raymond et al., 2010; Bravo et al., 2011; Visaggio et al., 2015). There may be indirect or direct relationship between bacterial virulence and other phenotypes, such as cell motility, biofilm formation and sporulation (Josenhans and Suerbaum, 2002; Gallego-Hernandez et al., 2020). In this study, we found that when *hfq* was deleted, the expression of many enterotoxin genes in bacteria, such as *nheA*, *nheB*, *entFM*, *entD*1, *entD*2, and *hbl*C, was significantly increased at the transcriptional level (Figure 6B), which to some extent enhanced bacterial virulence (de Maagd et al., 2003). In addition, the deletion of *hfq* also led to a significant increase in the amount of bacterial biofilm formation (Figure 3B), and the ability to form biofilms is also considered to be a key virulence factor in a variety of bacteria (Ramírez-Larrota and Eckhard, 2022). Environmental adaptability can help bacteria resist antibiotics and the host’s immune system, which facilitates bacterial survival and infection in the host, ultimately leading to host death (Al Safadi et al., 2012; Giaouris et al., 2015). Based on the above analysis, we believe that the increased insecticidal activity of the Δ3*hfq* strain may be the result of the combined effect of the increased expression level of enterotoxins and the enhanced biofilm formation ability.

Although how Hfq participates in the regulatory pathways of Bt has not been fully elucidated, our *hfq* deletion mutant Δ3*hfq* will provide new research materials for the application of Bt in bio-pesticides, and the effect of Hfq on Bt virulence is also important for increasing our understanding of the Bt insecticidal activity.

## Data Availability Statement

The datasets presented in this study can be found in online repositories. The names of the repository/repositories and accession number(s) can be found below: NCBI BioProject – PRJNA811318, SRP361911.

## Author Contributions

JH, ZY, and YF designed the experiments. ZY, YF, WZ, and WY did the experiments. JH, S-HC, ZY, and YF wrote and revised the manuscript. All authors contributed to the article and approved the submitted version.

## Conflict of Interest

The authors declare that the research was conducted in the absence of any commercial or financial relationships that could be construed as a potential conflict of interest.

## Publisher’s Note

All claims expressed in this article are solely those of the authors and do not necessarily represent those of their affiliated organizations, or those of the publisher, the editors and the reviewers. Any product that may be evaluated in this article, or claim that may be made by its manufacturer, is not guaranteed or endorsed by the publisher.

## Footnotes

1
http://hannonlab.cshl.edu/fastx_toolkit/

2
https://dbtbs.hgc.jp

3
http://kobas.cbi.pku.edu.cn
