# Supplementary material for: The RNA Chaperone Protein Hfq Regulates the Characteristic Sporulation and Insecticidal Activity of Bacillus thuringiensis
Source: Front Microbiol. 2022 Apr 11;13:884528. doi: 10.3389/fmicb.2022.884528 (PMC9037596; doi:10.3389/fmicb.2022.884528)
Supplement: Supplementary file 1 [file Data_Sheet_1.pdf]

The RNA chaperone protein Hfq regulates the characteristic sporulation and insecticidal activity of *Bacillus thuringiensis*

Zhaoqing Yu[1,#], Yang Fu[1,2,#], Wei Zhang[1], Li Zhu[1], Wen Yin[1], Shan-Ho Chou[1], Jin He[1,*]

[1]State Key Laboratory of Agricultural Microbiology, College of Life Science and Technology, Huazhong Agricultural University, Wuhan 430070, Hubei, China.

[2]National Engineering Research Center of Edible Fungi, Institute of Edible Fungi, Shanghai Academy of Agricultural Sciences, Shanghai 201403, China.

*** Correspondence:**

Jin He

hejin@mail.hzau.edu.cn

[#]These authors contributed equally to this work.

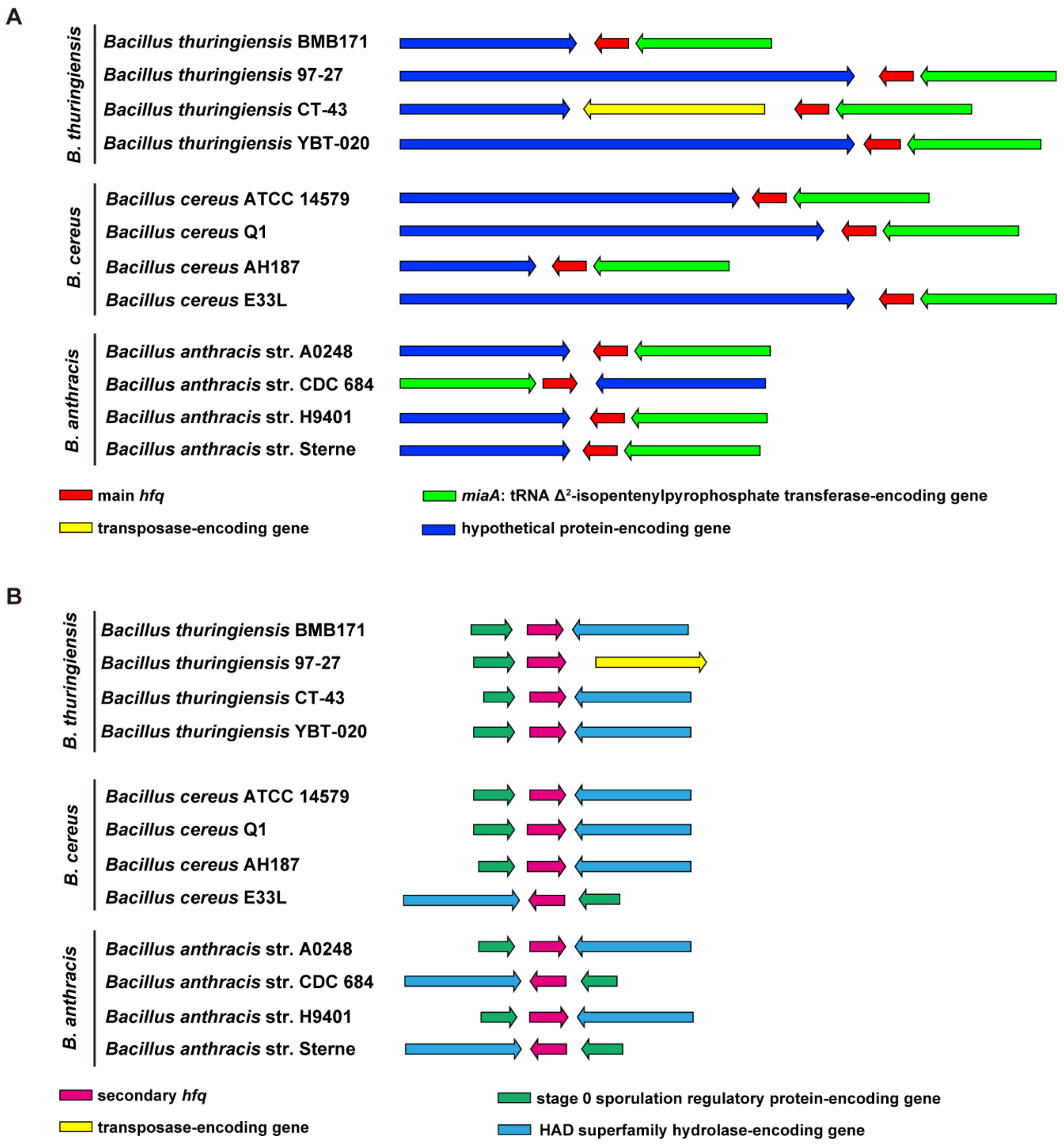

Figure S1. Genes flanking the two *hfq* gene copies commonly found in some completely sequenced genomes in the Bc group strains. The flanking genes of the main *hfq* gene copy **(A)** and another *hfq* gene copy **(B)** are relatively conserved.

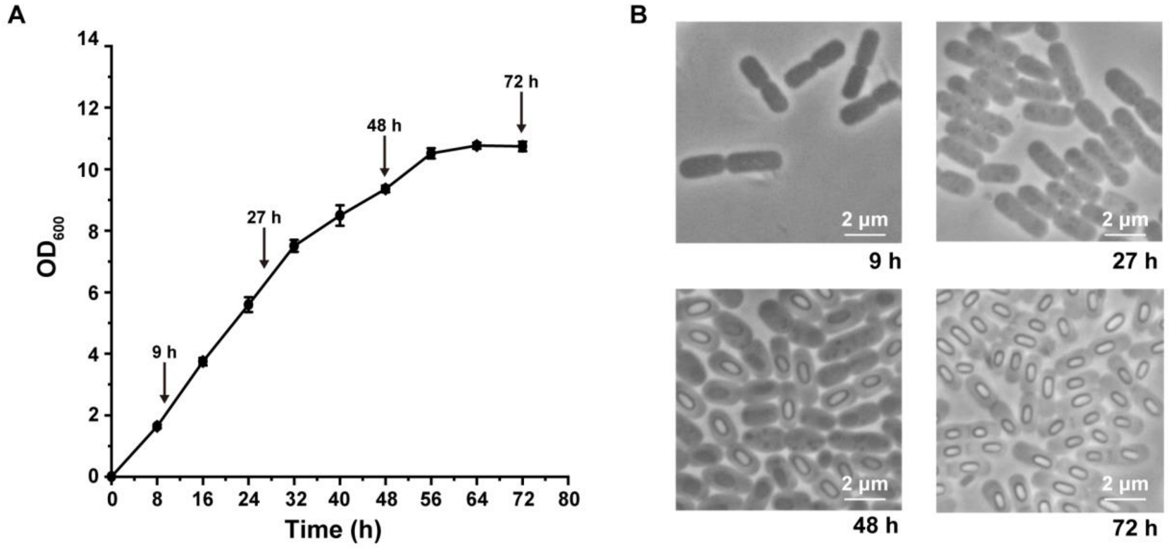

Figure S2. Growth cycle of Bt BMB171. **(A)** Growth curve of BMB171 over 72 h, with arrows indicating the time points of bacterial collection for cell morphology observation. **(B)** The cell morphology of BMB171 at 9 h, 27 h, 48 h and 72 h was observed by phase-contrast microscope, which confirmed that the growth phases of BMB171 at these four time points were logarithmic phase, early stationary phase, late stationary phase or early sporulation phase and final sporulation phase. The values are means ± SDs for triplicate assays.

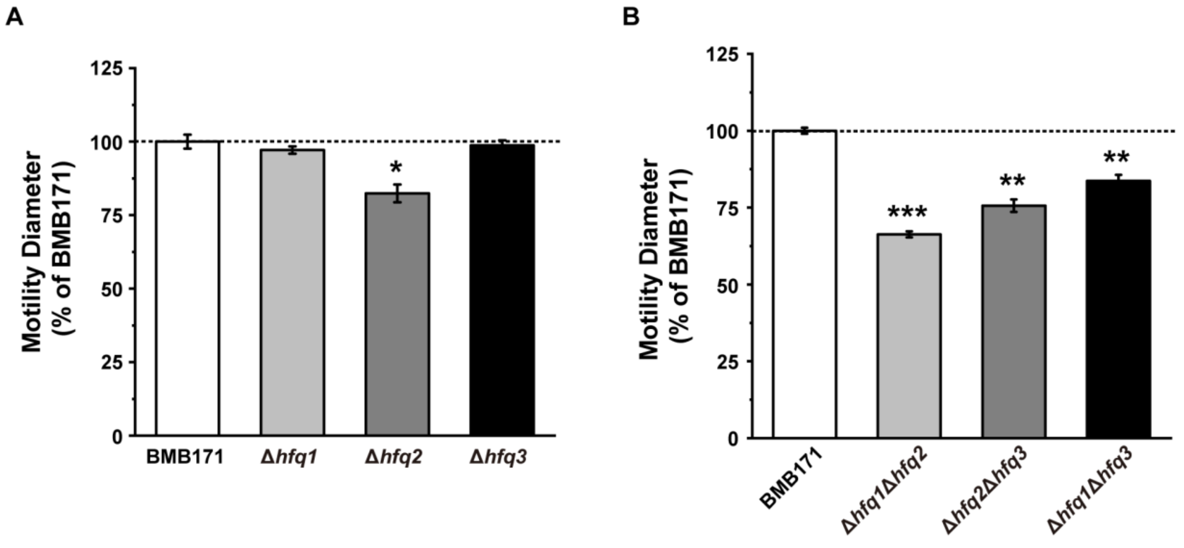

Figure S3. Motility comparison of all single-knockout and double-knockout strains of BMB171. **(A)** Colony diameters of BMB171, Δ*hfq1*, Δ*hfq2*, Δ*hfq3* on LB plates containing 0.5% agar. After comparing the different *hfq* single-knockout strains with the control strain BMB171, it is clear from the experimental results that the deletion of *hfq*2 exhibited a greater impact on bacterial motility. **(B)** Colony diameters of BMB171, Δ*hfq1hfq2*, Δ*hfq2hfq3*, and Δ*hfq1hfq3* on LB plates containing 0.5% agar. After comparing the *hfq* double-knockout strains with the *hfq* single-knockout strains, it can be seen from the experimental results that the bacterial motility was further inhibited as the *hfq* deletion copy numbers increased. Bacterial motility was maximally inhibited when *hfq*1 and *hfq*2 were knocked out at the same time. The values are means ± SDs for triplicate assays. Significances of differences by Student's t-test are indicated. ***$p < 0.001$; **$p < 0.01$; *$p < 0.05$.

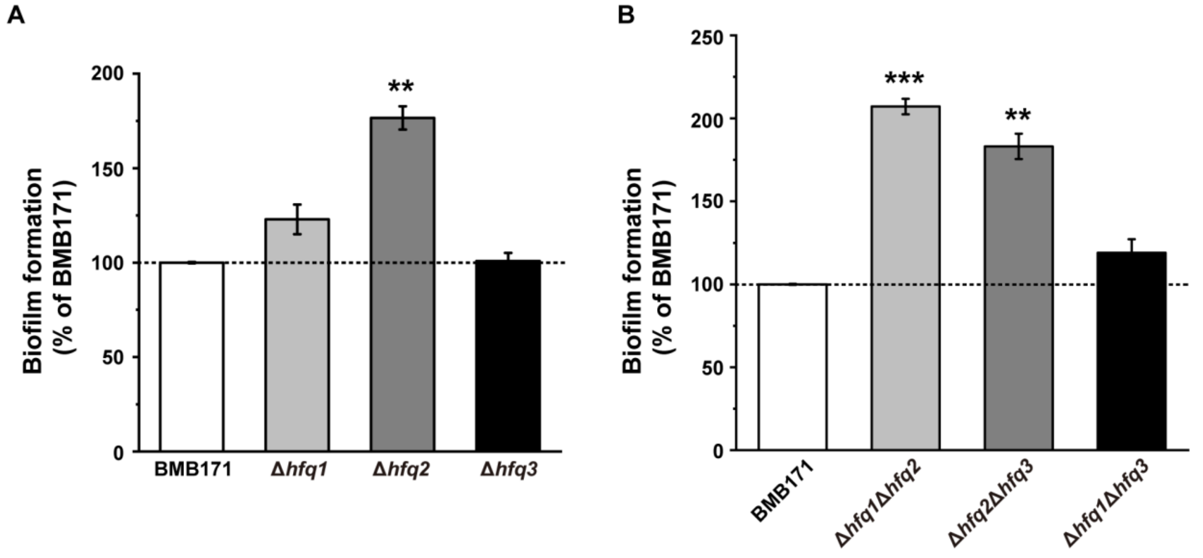

Figure S4. Biofilm formation quantification assays of BMB171 in single-knockout and double-knockout strains. **(A)** Quantification of biofilm formation for BMB171 and its *hfq* single knockout strains Δ*hfq1*, Δ*hfq2*, and Δ*hfq3* by crystal violet stain measured by UV spectrophotometer at 595 nm. The experimental results show that the deletion of *hfq2* greatly affected on the ability of bacterial biofilm formation. **(B)** Quantification of biofilm formation for BMB171 and its *hfq* double-knockout strains Δ*hfq1hfq2*, Δ*hfq2hfq3*, and Δ*hfq1hfq3* by crystal violet stain measured by UV spectrophotometer at 595 nm. It can be seen from the experimental results that the biofilm formation was enhanced in the *hfq* double knockout mutants. Yet, when *hfq1* and *hfq2* were knocked out at the same time, the biofilm formation is maximized. The values are means ± SDs for triplicate assays. Significances of differences by Student's t-test are indicated. ***$p < 0.001$; **$p < 0.01$; *$p < 0.05$.

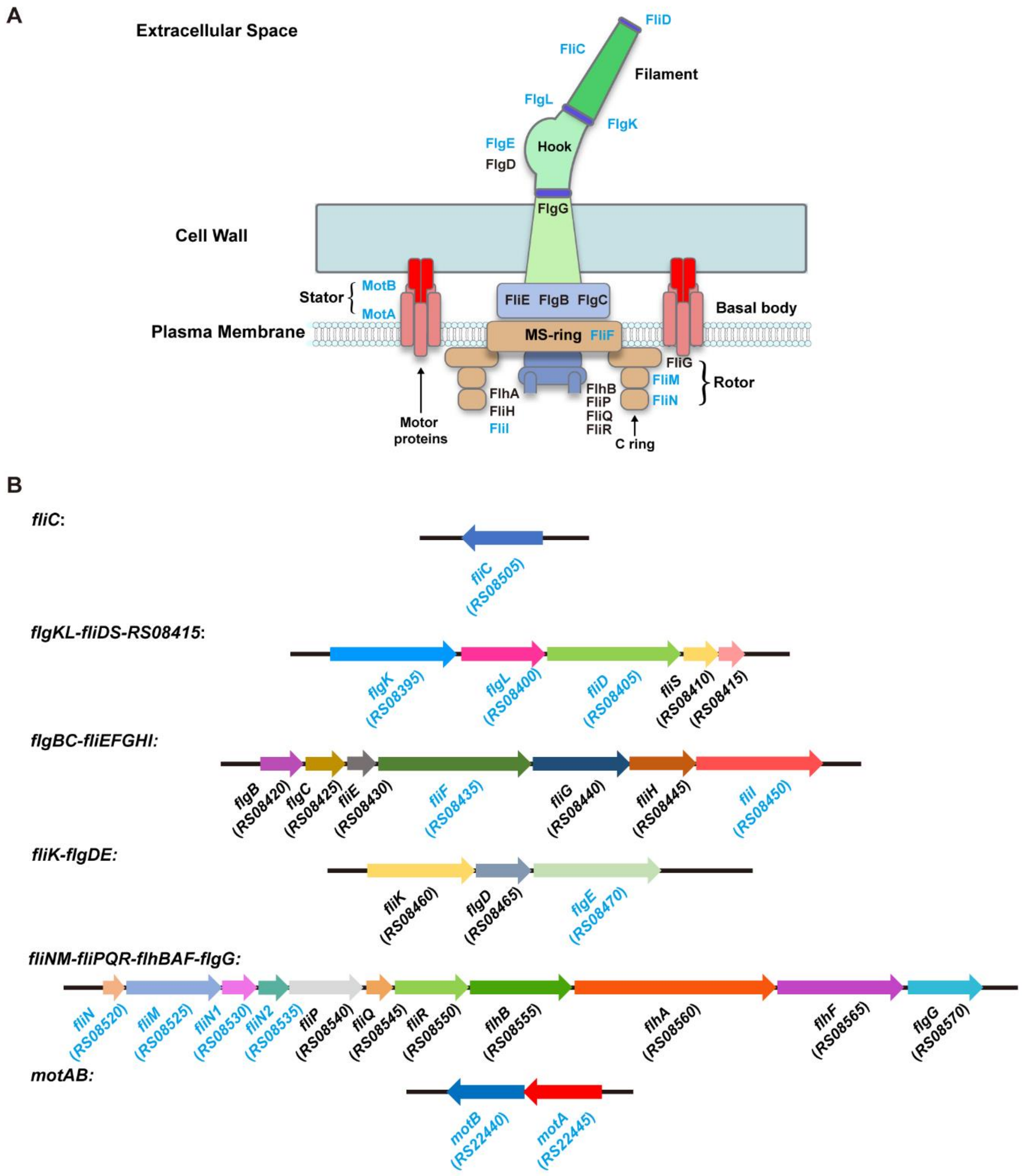

Figure S5. Flagellum and flagellum-associated genes in Bt BMB171. **(A)** Schematic representation of a bacterial flagellum. **(B)** Map location of the corresponding flagellum-associated genes. The prefix "BMB171" in each gene locus is omitted in this figure. The genes detected by RT-qPCR are marked in blue.

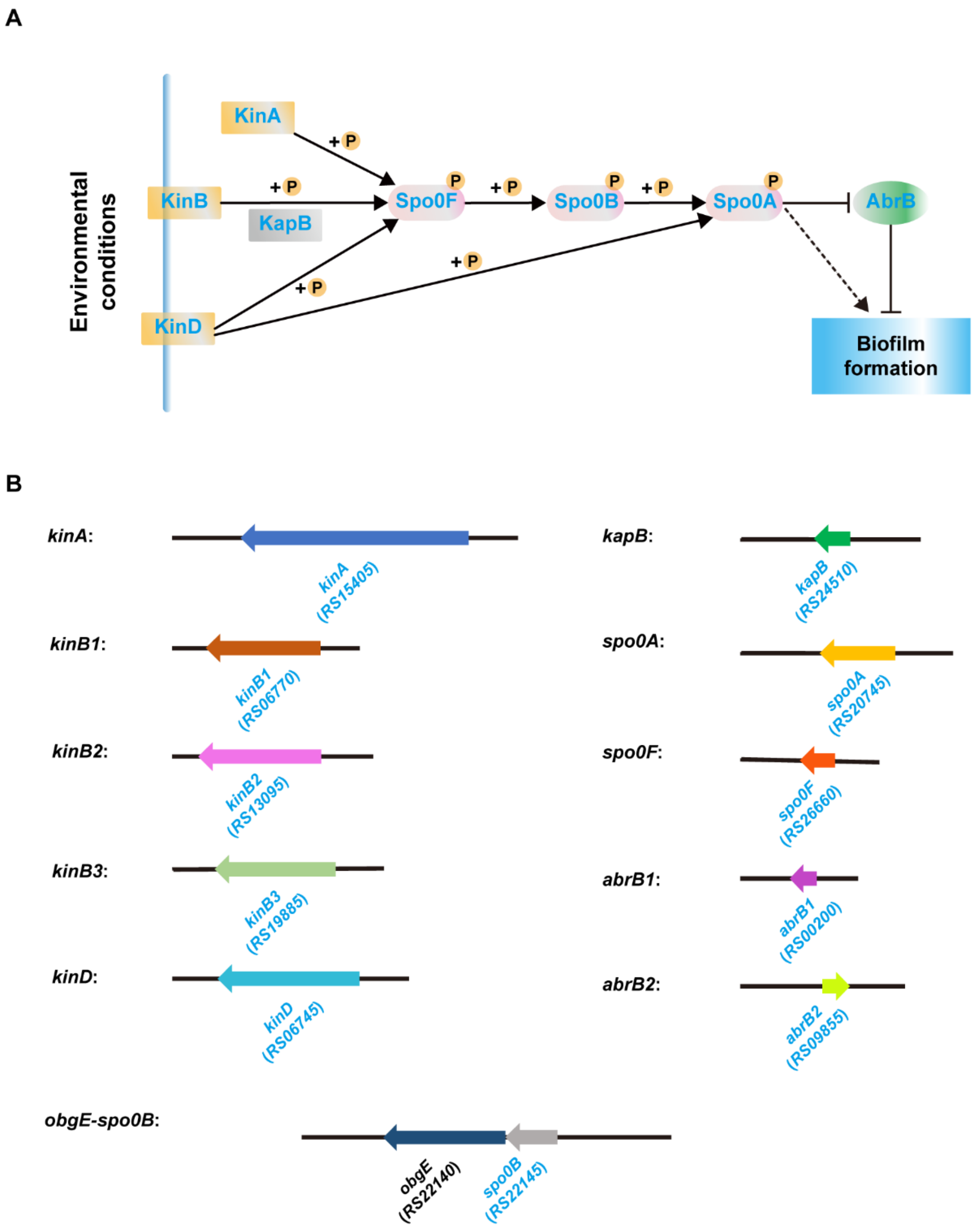

Figure S6. Biofilm formation regulatory pathway in Bt BMB171. **(A)** The pathway diagram of the regulation of biofilm formation in Bt BMB171, drawn according to the KEGG PATHWAY database (http://www.kegg.jp/kegg-bin/show_pathway?btb02020). **(B)** Map location of the corresponding biofilm formation-associated genes. The prefix "BMB171" in each gene locus is omitted in this figure. The genes detected by RT-qPCR are marked in blue.

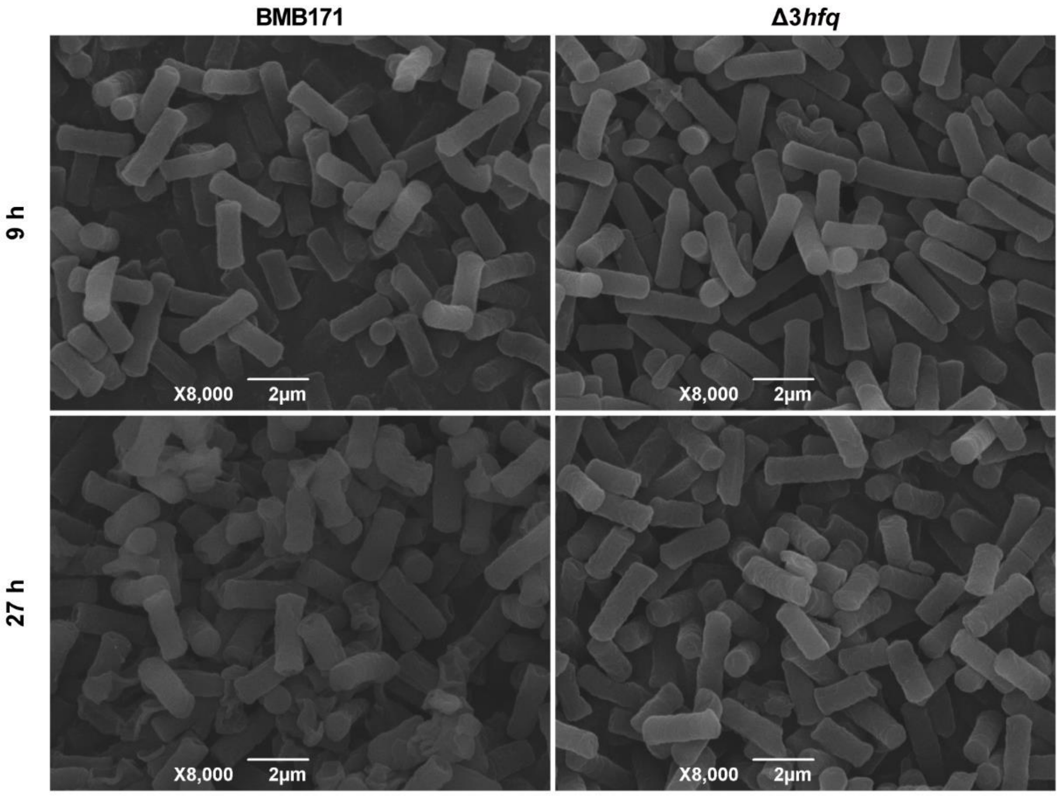

Figure S7. Cell morphology of BMB171 and Δ3*hfq*. Cell morphology of BMB171 and Δ3*hfq* as visualized by scanning electron microscope at 9 h and 27 h. The scale bar is 2 μm.

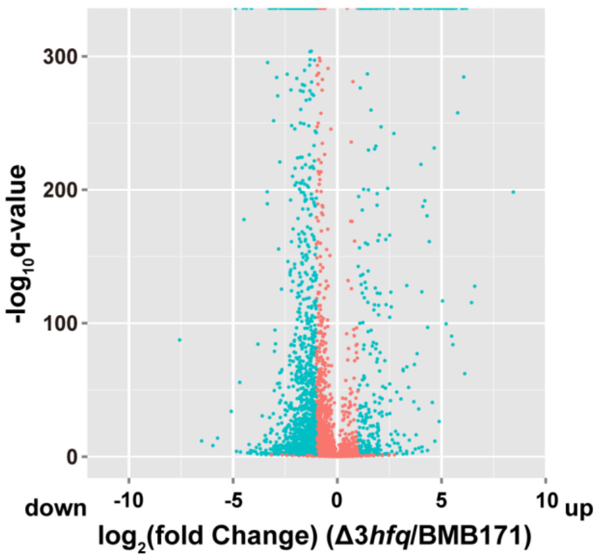

Figure S8. Volcano plot display of all gene expression for BMB171 and Δ3*hfq*. Blue dots represent each differentially expressed gene, and the red dots represent genes that are not differentially expressed.

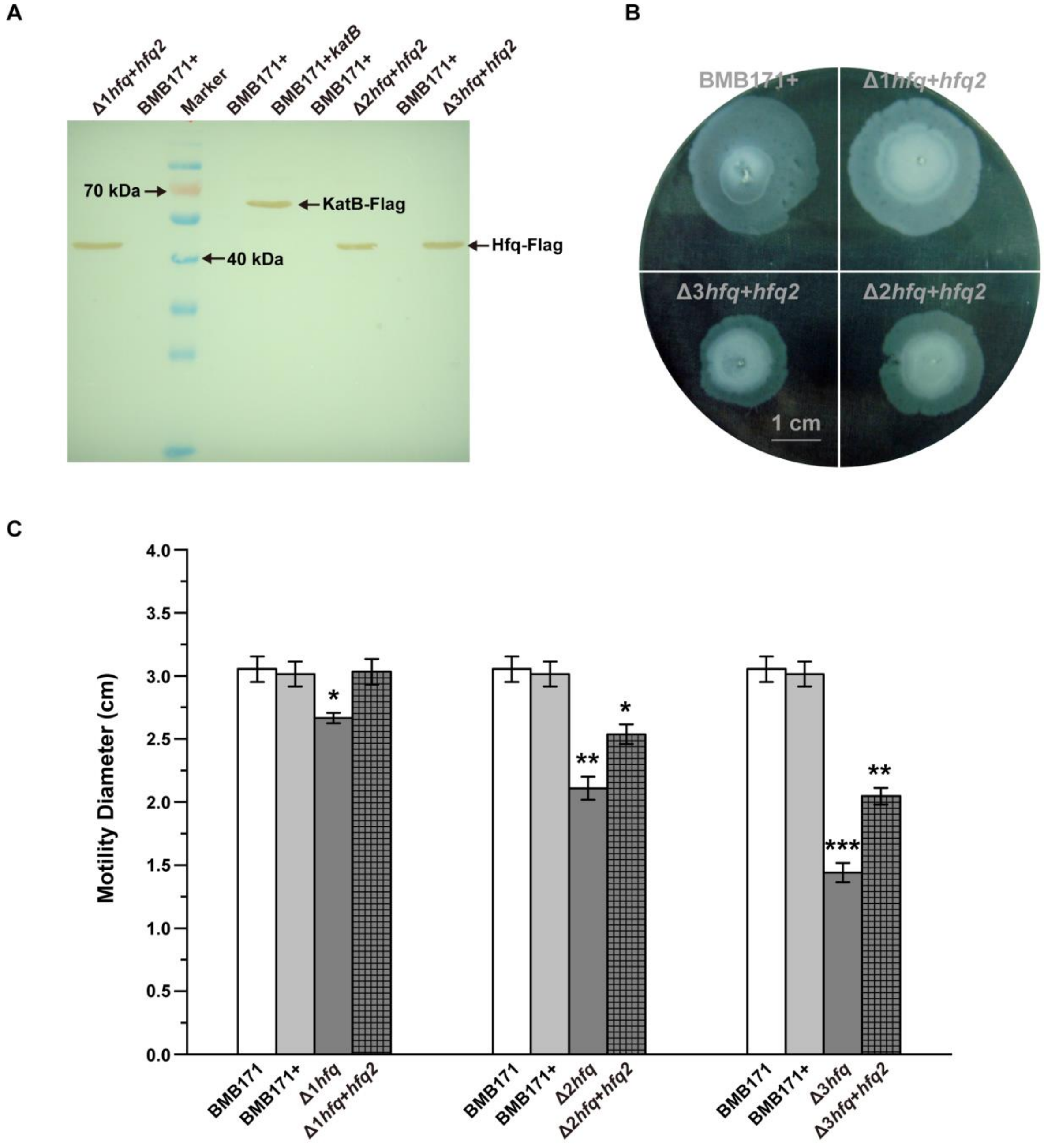

Figure S9. Complementation of the *hfq2* gene can restore the reduced motility in *hfq* deletion mutants. **(A)** Western blot detection of *hfq2*-complemented strains Δ1*hfq*+*hfq2* (lane 1), Δ2*hfq*+*hfq2* (lane 7), and Δ3*hfq*+*hfq2* (lane 9). A band corresponding to the size of the Hfq2 protein fused with a Flag-tag (Hfq2-Flag) could be detected in each complementing strain. Lanes 2, 4, and 6 all represent negative control (BMB171+), lane 3 is the prestained protein ladder (ThermoFisher Scientific, USA), and lane 5 represents positive control KatB-Flag. **(B)** Motility assay of BMB171+ and corresponding *hfq2*-complemented strains Δ1*hfq*+*hfq2*, Δ2*hfq*+*hfq2*, and Δ3*hfq*+*hfq2* on LB plates containing 0.5% agar. The scale bar is 1 cm. **(C)** Colony diameters

of BMB171, BMB171+, ,and *hfq* deletion mutants and corresponding *hfq2*-complemented strains. Complementation of the *hfq*2 gene can restore the reduced motility in *hfq* deletion mutants. Each group of experiments was repeated 3 times, and each sample was set up with 3 biological replicates.

Table S1. Sequences of oligonucleotide primers used in this study

Name	Sequence (5’ to 3’)	Reference
**Cloning primers for mutant construction**		
U*hfq1*F	CCCAAGCTTCATAAAGAGAGTTGAATGATATGGGAG	This work
U*hfq1*R	CCGACGCGTATATTGATTGATTGCTTCATGTCG	
D*hfq1*F	CCGACGCGTGAATTAGAATAGTAATGAATTATTGTAC	This work
D*hfq1*R	CGCGGATCCTGGCATAGGAGTTACTGGAC	
U*hfq2*F	CCCAAGCTTTCCCCAAGTACACTTAATGTTTG	This work
U*hfq2*R	CCGACGCGTTTACTGTCCTCTCCTTTCTGTGA	
D*hfq2*F	CCGACGCGTGACACTCTTGTTGAGTGTCTTAT	This work
D*hfq2*R	CGCGGATCCAATATCAAACTTCCTTTTTCTTC	
U*hfq3*F	CCCAAGCTTTTACAATAGATAAAGCAAGTGAGTA	This work
U*hfq3*R	CCGACGCGTCTAATTTCGTTTTCAATGAGTTC	
D*hfq3*F	CCGACGCGTAAAGCCCACGGACGGGAC	This work
D*hfq3*R	CGCGGATCCTCCAGCAAAAAGTCCTATATACG	
**Real Time PCR Primers**		
Q*hfq1*F	GAACATTTACAGGAAGAATTG	This work
Q*hfq1*R	TTTCATTATTGTTGATATAGCCTGC	
Q*hfq2*F	AGAAAATACGTTTGTTACGCTGGTACAAATGTGGAGATT	This work
Q*hfq2*R	GCATG	
Q*hfq3*F	GAAAACGAAATTACAATCATTTC	This work
Q*hfq3*R	GAATCGAATCGTGGAGATTGCATG	
Q*fliD*F	GCTCCACTTACTCCTTCAAAAACTC	This work
Q*fliD*R	AGTCGATGTGAAAAATAGTTGATCC	
Q*fliC*F	TACTGGTGACGCTGCTTTGGG	This work
Q*fliC*R	GGGAGGAGTGGCAGTTGTATCTAAG	
Q*flgL*F	AACATTCATTGGCAAATATGGAGC	This work
Q*flgL*R	ATCAATCTCTACACCAATAGCCTGC	
Q*flgK*F	TTACGAATCTGCTGGAGGGAG	This work
Q*flgK*F	TTTTTACCTGTCGTTCCTACCATAG	
Q*flgE*F	GAGACAGCAATAGAGGGAAATGG	This work
Q*flgE*R	ATTGGAATATGAATTGGACTTGGTC	
Q*fliF*F	AAAAGAAATGAATGGGATGGGC	This work
Q*fliF*R	CAATGTAATTTGAACATTTGCCG	
Q*fliI*F	TTTTGCTGGGTCAGGTGTTGG	This work
Q*fliI*R	GATGACTTTCATCTGACGTCGCTAC	
Q*fliM*F	AATGTTGGCTTGGCGGAGATAG	This work
Q*fliM*R	TGATATCGCTCGCTGTCGTAATC	

Q*fliN*F	TGAAGCATGAAGTATCTCCTGTGTC	This work
Q*fliN*R	TCTACTTCAAGAACATCGCCAAC	
Q*motA*F	TATGAAATTTGTGATTGATGGCG	This work
Q*motA*R	TAGCATGACCTAACTTTTCAATATCAG	
Q*motB*F	TTACTTGTTTCAGCGTCACCTCG	This work
Q*motB*R	GTATTCTCCGTATCCAATCGCAC	
Q*kinB1*F	GCCTATTGCTGGTGGTTCTATCTTA	This work
Q*kinB1*R	TTGAAAGGAAGAATGGTATCCAACT	
Q*kinB2*F	CCCTTTGTTCTAGTATTCCGCTTAT	This work
Q*kinB2*R	GCTGTAACGATGAACATTATTCCGT	
Q*kinB3*F	GAAATGCCTCCGAAGCCAAAT	This work
Q*kinB3*R	AAAACTGGAAGAAAGCTCATTCCCT	
Q*kinD*F	TATTGCTTGTTATCCTACCGACAAT	This work
Q*kinD*R	TTACCAGTCTCGGTGCCTACAAC	
Q*kinA*F	TCGAAAAGCTCTATGGAGGGAT	This work
Q*kinA*R	GAGGTTCCCAATACTCTCCAATG	
Q*kapB*F	AAATTGGCGAAATCGTTACTGGT	This work
Q*kapB*R	ATGTTCGTCTGTTCACGGAAAGC	
Q*spo0F*F	GAAGGTTATCAGACGTTCCAAGC	This work
Q*spo0F*R	GGATCATATCAAGCTCTCCATAAGC	
Q*spo0B*F	TTCAAGACATGATTGGCTCAATC	This work
Q*spo0B*R	TGTAATTTCCCTAATACTTCGTACTCC	
Q*spo0A*F	CATCATTCCGATCAGCAACAAC	This work
Q*spo0A*R	GCGATATCTGGATACAATACTTTCG	

Underlined: restriction sites F: Forward primers R: Reverse primers

U: upstream/upstream arms D: downstream/downstream arms

M: primer location in the middle of genes Q: RT-qPCR

Table S2. Genes flanking the main *hfq* gene in some completely sequenced genomes in the genus *Bacillus* strains

**Strains**		**Description**	**Length (bp)**	**Location**
**genus *Bacillus***				
***Bacillus cereus* group**				
Bacillus thuringiensis				
Bacillus thuringiensis BMB171	upstream gene	hypothetical protein-encoding gene	1197	3601175..3602371
	hfq	RNA-binding protein	225	complement(3602453..3602677)
	downstream gene	*miaA*	954	complement(3602699..3603652)
Bacillus mycoides				
Bacillus mycoides BPN36-3	upstream gene	*miaA*	954	4489086..4490039
	hfq	RNA-binding protein	225	4490061..4490285
	downstream gene	collagen-like protein gene	3627	complement(4490366..4493992)
Bacillus cereus				
Bacillus cereus ATCC 14579	upstream gene	hypothetical protein-encoding gene	2643	3678077..3680719
	hfq	RNA-binding protein	225	complement(3680801..3681025)
	downstream gene	*miaA*	954	complement(3681047..3682000)
Bacillus anthracis				
Bacillus anthracis str. A0248	upstream gene	hypothetical protein-encoding gene	1437	3514629..3516065
	hfq	RNA-binding protein	225	complement(3516146..3516370)
	downstream gene	*miaA*	954	complement(3516392..3517345)
Bacillus wiedmannii				
Bacillus wiedmannii str. SR52	upstream gene	aspartyl-phosphate phosphatase Spo0E family protein gene	219	1570764..1570982
	hfq	RNA-binding protein	189	1571033..1571221
	downstream gene	HD domain-containing protein gene	603	complement(1571247..1571849)
Bacillus manliponensis				

Bacillus manliponensis str. JCM 15802	upstream gene	*miaA*	954	14356..15309
	hfq	RNA-binding protein	225	15331..15555
	downstream gene	hypothetical protein-encoding gene	957	complement(15586..16542)
Bacillus tropicus				
Bacillus tropicus str. AOA-CPS1	upstream gene	hypothetical protein-encoding gene	3606	2966867..2970472
	hfq	RNA-binding protein	225	complement(2970553..2970777)
	downstream gene	*miaA*	954	complement(2970799..2971752)
Bacillus cytotoxicus				
Bacillus cytotoxicus str. CH_13	upstream gene	hypothetical protein-encoding gene	2529	2468194..2470722
	hfq	RNA-binding protein	225	complement(2470816..2471040)
	downstream gene	*miaA*	951	complement(2471066..2472016)
Bacillus toyonensis				
Bacillus toyonensis str. P18	upstream gene	IS3 family transposase-encoding gene	1355	3539143..3540497
	hfq	RNA-binding protein	225	complement(3540573..3540797)
	downstream gene	*miaA*	954	complement(3540819..3541772)
Bacillus pseudomycoides				
Bacillus pseudomycoides DSM 12442	upstream gene	*miaA*	951	3043171..3044121
	hfq	RNA-binding protein	225	3044147..3044371
	downstream gene	hypothetical protein-encoding gene	499	complement(3044442..3044940)
Bacillus paranthracis				
Bacillus paranthracis str. MN1F	upstream gene	*miaA*	954	3607953..3608906
	hfq	RNA-binding protein	219	3608928..3609146
	downstream gene	hypothetical protein-encoding gene	1455	complement(3609232..3610686)
Bacillus albus				
Bacillus albus str. PFYN01	upstream gene	*miaA*	954	1652834..1653787
	hfq	RNA-binding protein	225	1653809..1654033

	downstream gene	IS3 family transposase-encoding gene	1301	complement(1654109..1655409)
Bacillus bombysepticus				
Bacillus bombysepticus str. Wang	upstream gene	hypothetical protein-encoding gene	3138	3427283..3430420
	hfq	RNA-binding protein	225	complement(3430501..3430725)
	downstream gene	*miaA*	954	complement(3430747..3431700)
Bacillus pacificus				
Bacillus pacificus str. anQ-h4	upstream gene	*miaA*	954	1631688..1632641
	hfq	RNA-binding protein	225	1632663..1632887
	downstream gene	hypothetical protein-encoding gene	1653	complement(1632968..1634620)
Bacillus luti				
Bacillus luti str. FJ	upstream gene	*miaA*	954	1635610..1636563
	hfq	RNA-binding protein	225	1636585..1636809
	downstream gene	hypothetical protein-encoding gene	2976	complement(1636890..1639865)
Bacillus clarus				
Bacillus clarus str. BHP DJ93	upstream gene	hypothetical protein-encoding gene	423	complement(38892..39314)
	hfq	RNA-binding protein	204	39595..39798
	downstream gene	hypothetical protein-encoding gene	339	39841..40179
Bacillus nitratireducens				
Bacillus nitratireducens str. BM02	upstream gene	hypothetical protein-encoding gene	1329	3450481..3451809
	hfq	RNA-binding protein	225	complement(3451890..3452114)
	downstream gene	*miaA*	951	complement(3452140..3453090)
Bacillus gaemokensis				
Bacillus gaemokensis str. KCTC 13318	upstream gene	IS110 family transposase-encoding gene	156	complement(161130..>161285)
	hfq	RNA-binding protein	173	complement(161466..161638)
	downstream gene	FAD-dependent monooxygenase-encoding gene	1635	161975..163609

Bacillus paramycoides				
Bacillus paramycoides str. DE0103	upstream gene	DUF6442 family protein-encoding gene	306	complement(8415..8720)
	hfq	RNA-binding protein	189	complement(9377..9565)
	downstream gene	transposase-encoding gene	336	complement(9593..9928)
Bacillus proteolyticus				
Bacillus proteolyticus str. TD42	upstream gene	serine hydrolase-encoding gene	1521	7280..8800
	hfq	RNA-binding protein	189	9093..9281
	downstream gene	hypothetical protein-encoding gene	174	10281..10454
Bacillus mobilis				
Bacillus mobilis str. ML-A2C4	upstream gene	Spo0E family protein-encoding gene	219	1647482..1647700
	hfq	RNA-binding protein	189	1647754..1647942
	downstream gene	HD domain-containing protein-encoding gene	603	complement(1647970..1648572)
***Bacillus subtilis* group**				
Bacillus sonorensis				
Bacillus sonorensis J41TS2	upstream gene	*miaA*	945	495550..496494
	hfq	RNA-binding protein	222	496535..496756
	downstream gene	*ymzA*	225	496915..497139
Bacillus licheniformis				
Bacillus licheniformis str. SCDB 14	upstream gene	hypothetical protein-encoding gene	213	complement(2394699..2394911)
	hfq	RNA-binding protein	222	complement(2395202..2395423)
	downstream gene	*miaA*	945	complement(2395465..2396409)
Bacillus atrophaeus				
Bacillus atrophaeus subsp. *globigii*	upstream gene	*miaA*	945	1253399..1254343
	hfq	RNA-binding protein	222	1254383..1254604

	downstream gene	hypothetical protein-encoding gene	267	1254723..1254989
Bacillus subtilis				
Bacillus subtilis str. 168	upstream gene	*miaA*	945	1866389..1867333
	hfq	RNA-binding protein	222	1867373..1867594
	downstream gene	*ymzC*	273	1867790..1868062
Bacillus velezensis				
Bacillus velezensis str. JS25R	upstream gene	*miaA*	945	1795594..1796538
	hfq	RNA-binding protein	222	1796578..1796799
	downstream gene	hypothetical protein-encoding gene	276	1796897..1797172
Bacillus amyloliquefaciens				
Bacillus amyloliquefaciens IT-45	upstream gene	*miaA*	276	complement(2118647..2118922)
	hfq	RNA-binding protein	222	complement(2119019..2119240)
	downstream gene	hypothetical protein-encoding gene	945	complement(2119280..2120224)
Bacillus paralicheniformis				
Bacillus paralicheniformis str. NCTC8721	upstream gene	*miaA*	945	1951908..1952852
	hfq	RNA-binding protein	222	1952893..1953114
	downstream gene	hypothetical protein-encoding gene	213	1953428..1953640
**Other bacterial species**				
Bacillus glycinifermentans				
Bacillus glycinifermentans str. SRCM103574	upstream gene	*miaA*	945	2125463..2126407
	hfq	RNA-binding protein	222	2126448..2126669
	downstream gene	hypothetical protein-encoding gene	213	2126820..2127032
Bacillus weihaiensis				
Bacillus weihaiensis str. Alg07	upstream gene	*spoVK*	993	complement(2228779..2229771)
	hfq	RNA-binding protein	222	complement(2230441..2230662)

	downstream gene	*miaA*	939	complement(2230702..2231640)
Bacillus solimangrovi				
Bacillus solimangrovi str. GH2-4	upstream gene	hypothetical protein-encoding gene	963	64404..65366
	hfq	RNA-binding protein	225	complement(65468..65692)
	downstream gene	*miaA*	930	complement(65740..66669)
Bacillus mesophilus				
Bacillus mesophilus str. SA4	upstream gene	*spoVK*	951	complement(36256..37206)
	hfq	RNA-binding protein	234	complement(37462..37695)
	downstream gene	*miaA*	936	complement(37734..38669)
Bacillus carboniphilus				
Bacillus carboniphilus str. SaN35-3	upstream gene	*spoVK*	936	complement(46442..47377)
	hfq	RNA-binding protein	225	complement(47707..47931)
	downstream gene	*miaA*	957	complement(47962..48918)
Bacillus timonensis				
Bacillus timonensis str. MM10403188	upstream gene	*spoVK*	927	complement(590317..591243)
	hfq	RNA-binding protein	225	complement(591764..591988)
	downstream gene	*miaA*	948	complement(592081..593028)
Bacillus marinisedimentorum				
Bacillus marinisedimentorum str. NC2-31	upstream gene	*miaA*	948	117038..117985
	hfq	RNA-binding protein	228	118029..118256
	downstream gene	*ymzA*	1002	complement(118309..119310)
Bacillus sinesaloumensis				
Bacillus sinesaloumensis str. Marseille-P3516T	upstream gene	*miaA*	939	1060691..1061629
	hfq	RNA-binding protein	225	1061721..1061945
	downstream gene	*spoVK*	945	1062370..1063314
Bacillus taeanensis				

Bacillus taeanensis str. BH030017	upstream gene	hypothetical protein-encoding gene	426	299269..299694
	hfq	RNA-binding protein	204	299819..300022
	downstream gene	galactose-1-epimeraseencoding gene	1047	300244..301290
Bacillus tianshenii				
Bacillus tianshenii str. DSM 25879	upstream gene	*miaA*	951	197432..198382
	hfq	RNA-binding protein	228	198413..198640
	downstream gene	*spoVK*	933	198935..199867
Bacillus pumilus				
Bacillus pumilus str. 3-19	upstream gene	hypothetical protein-encoding gene	333	complement(2534955..2535287)
	hfq	RNA-binding protein	222	complement(2535448..2535669)
	downstream gene	*miaA*	957	complement(2535695..2536651)
Bacillus weihaiensis				
Bacillus weihaiensis str. Alg07	upstream gene	*spoVK*	993	complement(2228779..2229771)
	hfq	RNA-binding protein	222	complement(2230441..2230662)
	downstream gene	*miaA*	939	complement(2230702..2231640)
Bacillus dakarensis				
Bacillus dakarensis str. Marseille-P3515T	upstream gene	*miaA*	963	1410147..1411109
	hfq	RNA-binding protein	252	1411138..1411389
	downstream gene	*spoVK*	948	1411705..1412652
Bacillus alveayuensis				
Bacillus alveayuensis str. 24KAM51 LG50_119	upstream gene	*spoVK*	930	complement(70139..71068)
	hfq	RNA-binding protein	225	complement(71364..71588)
	downstream gene	*miaA*	948	complement(71622..72569)
Bacillus acidicola				
Bacillus acidicola str. FJAT-2406	upstream gene	*spoVK*	945	complement(1552296..1553240)
	hfq	RNA-binding protein	237	complement(1553878..1554114)

	downstream gene	*miaA*	957	complement(1554141..1555097)
Bacillus alkalicellulosilyticus				
Bacillus alkalicellulosilyticus str. FJAT-44921	upstream gene	hypothetical protein-encoding gene	978	3029470..3030447
	hfq	RNA-binding protein	237	complement(3030561..3030797)
	downstream gene	*miaA*	978	complement(3030794..3031771)
Bacillus stratosphericus				
Bacillus stratosphericus str. LK33	upstream gene	hypothetical protein-encoding gene	333	complement(29350..29682)
	hfq	RNA-binding protein	222	complement(29845..30066)
	downstream gene	*miaA*	951	complement(30092..31042)
Bacillus aerophilus				
Bacillus aerophilus str. KJ82	upstream gene	*miaA*	951	1693592..1694542
	hfq	RNA-binding protein	222	1694568..1694789
	downstream gene	YmzC family protein-encoding gene	333	1694952..1695284
Bacillus infantis				
Bacillus infantis NRRL B-14911	upstream gene	*miaA*	1002	1891643..1892644
	hfq	RNA-binding protein	234	1892673..1892906
	downstream gene	hypothetical protein-encoding gene	276	complement(1893232..1893507)
Bacillus smithii				
Bacillus smithii str. DSM 4216	upstream gene	*spoVK*	957	1752835..1753791
	hfq	RNA-binding protein	237	complement(1754109..1754345)
	downstream gene	*miaA*	945	complement(1754383..1755327)
Bacillus coahuilensis				
Bacillus coahuilensis m2-6 ABFM000025	upstream gene	*miaA*	930	54089..55018
	hfq	RNA-binding protein	228	55065..55292
	downstream gene	*spoVK*	990	55522..56511
Bacillus yapensis				

Bacillus yapensis str. XXST-01	upstream gene	*miaA*	966	187591..188556
	hfq	RNA-binding protein	240	188583..188822
	downstream gene	*spoVK*	948	189137..190084
Bacillus badius				
Bacillus badius str. NBPM-293	upstream gene	*miaA*	945	1336295..1337239
	hfq	RNA-binding protein	234	1337277..1337510
	downstream gene	*spoVK*	942	1337677..1338618
Bacillus aerolatus				
Bacillus aerolatus str. CX253	upstream gene	*miaA*	972	350880..351851
	hfq	RNA-binding protein	234	351883..352116
	downstream gene	*spoVK*	936	352281..353216
Bacillus canaveralius				
Bacillus canaveralius str. ATCC 29669	upstream gene	*miaA*	963	11355..12317
	hfq	RNA-binding protein	237	12345..12581
	downstream gene	*spoVK*	948	13009..13956
Bacillus wudalianchiensis				
Bacillus wudalianchiensis str. FJAT-27215	upstream gene	*spoVK*	936	complement(79263..80198)
	hfq	RNA-binding protein	234	complement(80364..80597)
	downstream gene	*miaA*	954	complement(80637..81590)
Bacillus xiapuensis				
Bacillus xiapuensis str. FJAT-46582	upstream gene	*miaA*	945	1247758..1248702
	hfq	RNA-binding protein	234	1248740..1248973
	downstream gene	hypothetical protein-encoding gene	1326	1249076..1250401
Bacillus haikouensis				
Bacillus haikouensis str. MNJ12	upstream gene	*miaA*	957	2030676..2031632
	hfq	RNA-binding protein	234	2031662..2031895

	downstream gene	*spoVK*	954	2032255..2033208
Bacillus ectoiniformans				
Bacillus ectoiniformans str. DSM 28970	upstream gene	*miaA*	945	48439..49383
	hfq	RNA-binding protein	234	49423..49656
	downstream gene	hypothetical protein-encoding gene	1335	49795..51129
Bacillus massilionigeriensis				
Bacillus massilionigeriensis str. Marseille-P2348T	upstream gene	*spoVK*	948	complement(1237043..1237990)
	hfq	RNA-binding protein	237	complement(1238297..1238533)
	downstream gene	*miaA*	954	complement(1238571..1239524)
Bacillus tepidiphilus				
Bacillus tepidiphilus str. SYSU G01002	upstream gene	*miaA*	954	124359..125312
	hfq	RNA-binding protein	234	125341..125574
	downstream gene	*spoVK*	963	125968..126930
Bacillus oleivorans				
Bacillus oleivorans str. JC228	upstream gene	*miaA*	951	38753..39703
	hfq	RNA-binding protein	228	39751..39978
	downstream gene	cytochrome P450-encoding gene	1251	40163..41413
Bacillus testis				
Bacillus testis str. SIT10	upstream gene	*miaA*	963	1804155..1805117
	hfq	RNA-binding protein	228	1805143..1805370
	downstream gene	*spoVK*	939	1805740..1806678
Bacillus tuaregi				
Bacillus tuaregi str. Marseille-P2489T	upstream gene	*spoVK*	948	complement(2665519..2666466)
	hfq	RNA-binding protein	240	complement(2666714..2666953)
	downstream gene	*miaA*	963	complement(2666985..2667947)

Bacillus niameyensis				
Bacillus niameyensis str. SIT3	upstream gene	*spoVK*	942	complement(866904..867845)
	hfq	RNA-binding protein	228	complement(868036..868263)
	downstream gene	*miaA*	924	complement(868296..869219)
Bacillus gobiensis				
Bacillus gobiensis str. FJAT-4402	upstream gene	*miaA*	942	1149079..1150020
	hfq	RNA-binding protein	222	1150065..1150286
	downstream gene	*hutH*	1494	1150821..1152314
Bacillus capparidis				
Bacillus capparidis str. DSM 103394	upstream gene	*miaA*	942	579854..580795
	hfq	RNA-binding protein	222	580840..581061
	downstream gene	*hutH*	1494	581596..583089
Bacillus massiliglaciei				
Bacillus massiliglaciei str. Marseille-P2600T	upstream gene	*miaA*	954	738374..739327
	hfq	RNA-binding protein	234	739356..739589
	downstream gene	IS3 family transposase-encoding gene	580	complement(739662..>740241)
Bacillus aquiflavi				
Bacillus aquiflavi str. 3H-10	upstream gene	*miaA*	960	1275157..1276116
	hfq	RNA-binding protein	228	1276145..1276372
	downstream gene	*spoVK*	954	1276871..1277824
Bacillus salacetis				
Bacillus salacetis str. SKP7-4 34	upstream gene	*spoVK*	951	complement(3991..4941)
	hfq	RNA-binding protein	234	complement(5176..5409)
	downstream gene	*miaA*	942	complement(5449..6390)
Bacillus mesophilum				
Bacillus mesophilum str. IITR-54	upstream gene	*miaA*	969	558783..559751

	hfq	RNA-binding protein	234	559774..560007
	downstream gene	*spoVK*	951	560346..561296
Bacillus marasmi				
Bacillus marasmi str. Marseille-P3556	upstream gene	*miaA*	951	343664..344614
	hfq	RNA-binding protein	234	344643..344876
	downstream gene	*spoVK*	951	345019..345969
Bacillus benzoevorans				
Bacillus benzoevorans str. DSM 5391	upstream gene	*spoVK*	945	complement(120788..121732)
	hfq	RNA-binding protein	240	complement(122040..122279)
	downstream gene	*miaA*	960	complement(122315..123274)
Bacillus salipaludis				
Bacillus salipaludis str. WN066	upstream gene	*spoVK*	948	complement(63378..64325)
	hfq	RNA-binding protein	231	complement(64469..64699)
	downstream gene	*miaA*	957	complement(64726..65682)
Bacillus obstructivus				
Bacillus obstructivus str. VT-16-70	upstream gene	*spoVK*	942	complement(17615..18556)
	hfq	RNA-binding protein	228	complement(18949..19176)
	downstream gene	*miaA*	996	complement(19206..20201)
Bacillus massiliogorillae				
Bacillus massiliogorillae str. G2	upstream gene	*spoVK*	945	complement(2392566..2393510)
	hfq	RNA-binding protein	234	complement(2394106..2394339
	downstream gene	*miaA*	918	complement(2394375..2395292)
Bacillus methanolicus				
Bacillus methanolicus str. DE0127	upstream gene	*spoVK*	240	complement(19019..19258)
	hfq	RNA-binding protein	948	complement(17500..18447)
	downstream gene	*miaA*	990	complement(19284..20273)

Bacillus mediterraneensis				
Bacillus mediterraneensis str. Marseille-P2366T	upstream gene	*miaA*	954	1257703..1258656
	hfq	RNA-binding protein	231	1258682..1258912
	downstream gene	*spoVK*	948	1259078..1260025
Bacillus andreraoultii				
Bacillus andreraoultii str. SIT1	upstream gene	*spoVK*	945	complement(2036454..2037398)
	hfq	RNA-binding protein	225	complement(2037809..2038033)
	downstream gene	*miaA*	954	complement(2038063..2039016)
Bacillus solitudinis				
Bacillus solitudinis str. FJAT-45086	upstream gene	hypothetical protein-encoding gene	1002	253327..254328
	hfq	RNA-binding protein	231	complement(254376..254606)
	downstream gene	*miaA*	948	complement(254637..255584)
Bacillus andreraoultii				
Bacillus andreraoultii str. SIT1	upstream gene	*spoVK*	945	complement(2036454..2037398)
	hfq	RNA-binding protein	225	complement(2037809..2038033)
	downstream gene	*miaA*	954	complement(2038063..2039016)
Bacillus pakistanensis				
Bacillus pakistanensis str. DSM 24834	upstream gene	*miaA*	948	669596..670543
	hfq	RNA-binding protein	234	670574..670807
	downstream gene	*spoVK*	960	671148..672107
Bacillus rubiinfantis				
Bacillus rubiinfantis str. MGYG-HGUT-01475	upstream gene	*spoVK*	951	complement(93638..94588)
	hfq	RNA-binding protein	231	complement(94829..95059)
	downstream gene	*miaA*	957	complement(95088..96044)
Bacillus renqingensis				

Bacillus renqingensis str. REN2	upstream gene	*spoVK*	951	complement(130139..131089)
	hfq	RNA-binding protein	231	complement(131294..131524)
	downstream gene	*miaA*	957	complement(131551..132507)
Bacillus fonticola				
Bacillus fonticola str. CS13	upstream gene	*spoVK*	951	complement(1592869..1593819)
	hfq	RNA-binding protein	228	complement(1594018..1594245)
	downstream gene	*miaA*	945	complement(1594311..1595255)
Bacillus piscicola				
Bacillus piscicola str. FBU1786 2	upstream gene	*miaA*	300933	10062..310994
	hfq	RNA-binding protein	255	311044..311298
	downstream gene	hypothetical protein-encoding gene	990	complement(311335..312324)
Bacillus dafuensis				
Bacillus dafuensis str. FJAT-25496	upstream gene	*miaA*	969	2191816..2192784
	hfq	RNA-binding protein	231	2192813..2193043
	downstream gene	*spoVK*	948	2193296..2194243
Bacillus freudenreichi				
Bacillus freudenreichii str. NCTC4823	upstream gene	hypothetical protein-encoding gene	147	complement(2420280..2420426)
	hfq	RNA-binding protein	231	complement(2421017..2421247)
	downstream gene	*miaA*	978	complement(2421277..2422254)
Bacillus tamaricis				
Bacillus tamaricis str. CGMCC 1.15917 1578	upstream gene	hypothetical protein-encoding gene	1020	52731..53750
	hfq	RNA-binding protein	231	complement(53930..54160)
	downstream gene	*miaA*	927	complement(54208..55134)
Bacillus alkalicola				
Bacillus alkalicola str. JCM 17908 751	upstream gene	integrase-encoding gene	966	26419..27384
	hfq	RNA-binding protein	231	complement(27631..27861)

	downstream gene	*miaA*	927	complement(27906..28832)
Bacillus shivajii				
Bacillus shivajii str. JCM 32183	upstream gene	hypothetical protein-encoding gene	942	2508072..2509013
	hfq	RNA-binding protein	234	complement(2509074..2509307)
	downstream gene	*miaA*	921	complement(2509354..2510274)
Bacillus lacisalsi				
Bacillus lacisalsi str. YSP-3 ZB100000	upstream gene	*miaA*	918	1170262..1171179
	hfq	RNA-binding protein	231	1171227..1171457
	downstream gene	integrase-encoding gene	1035	complement(1171561..1172595)
Bacillus ndiopicus				
Bacillus ndiopicus str. FF3	upstream gene	*miaA*	906	969437..970342
	hfq	RNA-binding protein	228	970490..970717
	downstream gene	hypothetical protein-encoding gene	303	970766..971068
Bacillus cecembensis				
Bacillus cecembensis str. DSM 21993	upstream gene	*miaA*	900	102132..103031
	hfq	RNA-binding protein	228	103106..103333
	downstream gene	hypothetical protein-encoding gene	303	103403..103705
Bacillus safensis				
Bacillus safensis str. U14-5	upstream gene	*miaA*	957	3257615..3258571
	hfq	RNA-binding protein	222	3258597..3258818
	downstream gene	hypothetical protein-encoding gene	321	3258981..3259301
Bacillus thermozeamaize				
Bacillus thermozeamaize str. ZCTH02-B2 BIN02_NODE1312881525	upstream gene	*miaA*	969	42826..43794
	hfq	RNA-binding protein	261	43842..44102
	downstream gene	hypothetical protein-encoding gene	936	44274..45209

Bacillus fungorum				
Bacillus fungorum str. 17-SMS-01 ZB100003	upstream gene	hypothetical protein-encoding gene	219	268377..268595
	hfq	RNA-binding protein	189	268647..268835
	downstream gene	hypothetical protein-encoding gene	606	complement(268860..269465)

Table S3. The copy numbers of *hfq* genes from chromosome and plasmids in *Bacillus cereus* group

Strains	Chromosome	Plasmids	Total
B. anthracis str. A0248	2	1	3
B. anthracis str. Ames	2	0	2
B. anthracis str. CDC 684	2	1	3
B. anthracis str. H9401	3	0	3
B. anthracis str. Sterne	2	1	3
B. anthracis str. 'Ames Ancestor'	2	1	3
B. anthracis str. A16	2	1	3
B. anthracis str. A16R	2	1	3
B. anthracis str. SVA11	2	1	3
B. anthracis str. HYU01	2	1	3
B. anthracis str. Vollum	2	1	3
B. cereus 03BB102	2	1	3
B. cereus AH187	2	1	3
B. cereus AH820	2	1	3
B. cereus ATCC 10987	2	1	3
B. cereus ATCC 14579	2	0	2
B. cereus B4264	2	0	2
B. cereus E33L	2	1	3
B. cereus F837/76	2	0	2
B. cereus FRI-35	2	1	3
B. cereus G9842	2	2	4
B. cereus NC7401	2	1	3
B. cereus Q1	2	1	3
B. cereus biovar *anthracis* str. CI	2	1	3
B. cereus FT9	1	0	1
B. cytotoxicus NVH 391-98	1	0	1
B. mycoides Rock1-4	3	0	3
B. mycoides DSM 2048	2	0	2
B. mycoides 219298	4	0	4
B. mycoides ATCC 6462	2	0	2
B. pseudomycoides DSM 12442	4	0	4
B. thuringiensis Al Hakam	2	2	4
B. thuringiensis BMB171	2	1	3
B. thuringiensis Bt407	2	1	3
B. thuringiensis HD-771	2	4	6
B. thuringiensis HD-789	2	2	4

B. thuringiensis HD1011	2	1	3
B. thuringiensis MC28	1	2	3
B. thuringiensis CT-43	2	2	4
B. thuringiensis YBT-020	2	3	5
B. thuringiensis 97-27	3	0	3
B. thuringiensis serovar *thuringiensis* str. IS5056	2	3	5
B. thuringiensis YBT-1518	3	3	6
B. thuringiensis YBT-1520	2	1	3
B. thuringiensis serovar *kurstaki* str. HD-1	2	1	3
B. thuringiensis serovar *kurstaki* str. HD73	2	0	2
B. weihenstephanensis KBAB4	2	2	4
B. weihenstephanensis WSBC 10204	3	0	3

Table S4. Comparative analysis of the genes related to spore formation that controlled by the different sigma factor between BMB171 and Δ3*hfq*

Gene ID	Gene	Function	RPKM				log_2(fold change) (Δ3*hfq* *vs* BMB171)	*q*-vaule	Change (Δ3*hfq* *vs* BMB171)
			Δ3*hfq*-1	Δ3*hfq*-2	171-1	171-2			
**The expression level of genes controlled by SigH**									
BMB171_RS00255	*spoVG*	regulatory protein SpoVG	1073.7	1031.7	3785.8	5280.3	-2.09	0	down
BMB171_RS00565	*sigH*	RNA polymerase factor sigma-70	34.9	32.8	228.4	201.5	-2.68	3.56E-126	down
BMB171_RS01300	*yisK*	fumarylacetoacetase	544.6	532.5	2113.4	1979.7	-1.93	0	down
BMB171_RS04535	*yhaR*	enoyl-CoA hydratase	346.5	313.8	917.4	987.1	-1.53	0	down
BMB171_RS05835	*yisK*	fumarylacetoacetate hydrolase	76.4	83.7	50.2	47.7	0.71	5.88E-10	unchanged
BMB171_RS06485	*sinI*	SinI protein	82.6	81.5	216.7	241.1	-1.47	1.19E-13	down
BMB171_RS06750	*ykoM*	MarR family transcriptional regulator	3.3	3.8	4.6	7.0	-0.67	0.31165489	unchanged
BMB171_RS07015	*gltP*	proton/sodium-glutamate symport protein	14.4	15.6	16.5	17.1	-0.16	0.46070158	unchanged
BMB171_RS07240	*gltP*	proton/sodium-glutamate symport protein	21.9	21.6	16.9	14.9	0.44	0.01659589	unchanged
BMB171_RS07630	*hbs*	DNA-binding protein HU	1339.0	1476.3	3896.4	3309.5	-1.36	0	down
BMB171_RS07680	*ypiB*	hypothetical protein	657.9	654.8	2837.6	2725.5	-2.09	0	down
BMB171_RS07685	*ypiF*	hypothetical protein	215.1	203.6	1193.2	766.3	-2.25	0	down
BMB171_RS08030	*epsA*	capsular polysaccharide protein CpsC	52.3	50.3	172.8	171.7	-1.75	1.97E-66	down
BMB171_RS08035	*epsB*	tyrosine-protein kinase cpsD	58.6	64.3	188.4	161.6	-1.52	8.64E-47	down
BMB171_RS08040	*epsL*	undecaprenyl-phosphate galactosephosphotransferase	81.4	98.5	332.6	300.2	-1.82	1.32E-111	down
BMB171_RS08790	*citG*	fumarate hydratase	59.6	63.1	73.6	71.3	-0.24	0.00960047	unchanged
BMB171_RS08815	*yoeA*	Na^+ driven multidrug efflux pump	12.9	10.7	6.7	8.8	0.62	0.01150804	unchanged

BMB171_RS08840	*ccdA*	cytochrome c-type biogenesis protein	0.0	1.0	1.7	1.0	-1.42	0.24151446	unchanged
BMB171_RS08940	*gltP*	proton/sodium-glutamate symport protein	1.8	0.5	2.3	2.2	-1.01	0.11688924	unchanged
BMB171_RS09970	*ycsN*	IolS protein	10.7	15.6	16.7	16.1	-0.31	0.22166169	unchanged
BMB171_RS10480	*bsaA*	glutathione peroxidase	28.5	38.4	56.3	48.8	-0.65	0.0008331	unchanged
BMB171_RS10630	*spoVS*	stage V sporulation protein S	463.0	401.2	865.3	959.8	-1.08	3.24E-63	down
BMB171_RS10670	*ypgQ*	metal-dependent phosphohydrolase	12.0	6.4	13.7	15.5	-0.67	0.0410288	unchanged
BMB171_RS10695	*ypgR*	HEAT repeat-containing PBS lyase	221.0	227.7	209.9	203.5	0.12	0.04258701	unchanged
BMB171_RS11105	*racA*	polar chromosome segregation protein	292.8	259.7	1104.9	950.9	-1.91	0	down
BMB171_RS11205	*spo0M*	sporulation-control protein	126.3	124.6	315.1	332.1	-1.36	6.62E-89	down
BMB171_RS11470	*hbs*	DNA-binding protein HU	840.9	803.7	3054.1	3186.4	-1.92	0	down
BMB171_RS11480	*yisT*	DinB protein	7.1	10.5	24.2	25.1	-1.48	2.12E-06	down
BMB171_RS12185	*ynaD*	ribosomal-protein-serine acetyltransferase	24.2	21.7	28.6	30.1	-0.35	0.16364814	unchanged
BMB171_RS12350	*yhaR*	enoyl-CoA hydratase	215.2	238.0	439.0	463.0	-0.99	2.27E-77	unchanged
BMB171_RS13055	*kinA*	sporulation kinase	2.7	1.3	2.3	4.6	-0.76	0.07000268	unchanged
BMB171_RS14260	*bacF*	aspartate aminotransferase	41.8	39.8	73.9	70.7	-0.83	4.38E-15	unchanged
BMB171_RS15110	*yqxD*	hypothetical protein	6.7	8.6	16.8	17.3	-1.14	0.00372949	unchanged
BMB171_RS15405	*kinE*	sporulation kinase	11.7	10.9	19.8	24.3	-0.96	6.99E-12	unchanged
BMB171_RS18135	*ccdA*	cytochrome c-type biogenesis protein	6.3	10.2	14.6	14.7	-0.81	0.01061766	unchanged
BMB171_RS18475	*hbs*	DNA-binding protein HU	220.4	191.5	305.3	381.8	-0.73	4.15E-13	unchanged
BMB171_RS18590	*epsC*	UDP-N-acetylglucosamine 4,6-dehydratase	0.4	1.0	0.6	0.7	0.16	0.89689268	unchanged
BMB171_RS18715	*spoVS*	stage V sporulation protein S	1754.9	1780.1	1715.6	2303.3	-0.17	1.94E-05	unchanged
BMB171_RS19370	*ftsZ*	cell division protein FtsZ	303.3	315.0	567.1	658.4	-0.98	1.51E-149	unchanged
BMB171_RS19375	*ftsA*	cell division protein FtsA	319.8	306.8	703.6	788.2	-1.25	1.73E-304	down
BMB171_RS20035	*bacF*	transaminase	6.6	6.4	10.3	12.0	-0.77	0.00621356	unchanged
BMB171_RS20235	*sigF*	sporulation sigma factor SigF	3093.7	2964.5	12370.6	12571.2	-2.04	0	down
BMB171_RS20240	*spoIIAB*	anti-sigma F factor	1370.5	1336.6	6011.7	6088.9	-2.16	0	down

BMB171_RS20745	*spo0A*	stage 0 sporulation protein A	132.8	132.8	199.9	268.0	-0.80	4.26E-30	unchanged
BMB171_RS21410	*sigA*	RNA polymerase sigma factor RpoD	239.8	219.2	476.1	498.6	-1.09	7.79E-139	down
BMB171_RS21415	*dnaG*	DNA primase	201.6	200.0	649.0	574.5	-1.61	0	down
BMB171_RS21725	*vpr*	minor extracellular protease VpR	9.1	10.6	8.7	7.7	0.26	0.15636675	unchanged
BMB171_RS22180	*minD*	cell division inhibitor MinD	170.6	174.8	581.1	608.0	-1.78	1.02E-205	down
BMB171_RS22185	*minC*	septum formation inhibitor	468.8	439.5	1338.4	1420.5	-1.60	0	down
BMB171_RS22500	*yhaR*	enoyl-CoA hydratase	853.4	848.4	1824.3	1763.1	-1.08	0	down
BMB171_RS23150	*yttP*	transcriptional regulator IcaR	425.0	412.8	1050.9	918.0	-1.24	1.84E-197	down
BMB171_RS23290	*ytxJ*	hypothetical protein	55.7	52.6	111.2	81.5	-0.85	1.68E-06	unchanged
BMB171_RS23295	*ytxG*	general stress protein	65.9	86.3	119.7	127.8	-0.69	3.32E-09	unchanged
BMB171_RS24270	*glgP*	glycogen phosphorylase	157.1	173.6	607.3	571.0	-1.83	0	down
BMB171_RS24275	*glgA*	glycogen synthase	102.7	102.0	347.9	349.5	-1.77	5.91E-275	down
BMB171_RS24280	*glgD*	glucose-1-phosphate adenylyltransferase	121.0	98.7	509.2	426.9	-2.11	0	down
BMB171_RS24285	*glgC*	glucose-1-phosphate adenylyltransferase	193.8	198.1	701.1	686.3	-1.82	0	down
BMB171_RS24290	*glgB*	glycogen branching enzyme	213.3	201.4	599.9	579.2	-1.51	0	down
BMB171_RS24300	*vpr*	minor extracellular protease VpR	1.5	1.5	2.4	3.1	-0.86	0.0044817	unchanged
BMB171_RS25875	*yvyD*	sigma (54) modulation protein/SSU ribosomal protein S30P	4756.1	4640.3	6927.4	7855.1	-0.65	0	unchanged
BMB171_RS26325	*epsL*	undecaprenyl-phosphate galactosephosphotransferase	3.2	2.5	3.4	1.0	0.30	0.68146404	unchanged
BMB171_RS26340	*epsB*	tyrosine-protein kinase	3.2	7.3	11.8	14.3	-1.28	0.00042913	down
BMB171_RS26345	*epsA*	capsular polysaccharide biosynthesis chain length regulator	3.0	4.2	4.4	3.7	-0.18	0.76252165	unchanged
BMB171_RS26350	*epsB*	tyrosine-protein kinase	32.3	38.0	99.4	97.0	-1.48	3.02E-28	down
BMB171_RS26660	*spo0F*	sporulation initiation phosphotransferase F	134.9	110.9	243.5	265.6	-1.05	1.81E-23	down
**The expression level of genes controlled by SigF**									

BMB171_RS00155	*csfB*	CsfB protein	31.0	36.3	683.1	825.3	-4.47	2.80E-178	unchanged
BMB171_RS00220	*ksgA*	dimethyladenosine transferase	41.0	35.6	76.3	74.5	-0.98	2.53E-15	down
BMB171_RS00275	*subA*	hypothetical protein	155.2	111.6	279.8	335.5	-1.20	1.45E-21	down
BMB171_RS00285	*spoVT*	stage V sporulation protein T	498.1	551.4	1934.6	1895.3	-1.87	0	unchanged
BMB171_RS00500	*mcsB*	ATP:guanido phosphotransferase	711.9	658.1	1491.1	1222.6	-1.00	0	down
BMB171_RS00505	*clpC*	genetic competence negative regulator ClpC/MecB	1264.7	1289.9	3160.0	3119.7	-1.30	0	down
BMB171_RS00840	*gerD*	spore germination protein GerD	178.5	214.2	475.9	417.5	-1.19	1.54E-81	down
BMB171_RS01070	*yusW*	hypothetical protein	2813.1	2806.2	7512.7	6656.6	-1.34	0	down
BMB171_RS02110	*yhfW*	oxidoreductase	49.6	50.2	105.9	99.8	-1.05	2.61E-38	unchanged
BMB171_RS02580	*yfhE*	hypothetical protein	551.0	406.9	482.7	747.4	-0.34	0.00171694	unchanged
BMB171_RS02835	*yhbA*	iron-sulfur cluster-binding protein	15.0	16.8	23.0	22.1	-0.50	0.00832017	down
BMB171_RS03265	*gerAC*	spore germination protein KC	3.0	4.0	12.9	12.6	-1.87	2.56E-09	unchanged
BMB171_RS03270	*gerAB*	spore germination protein KB	0.3	2.5	3.2	3.1	-1.13	0.05516708	unchanged
BMB171_RS03275	*gerAA*	spore germination protein KA	10.6	10.5	20.1	15.4	-0.76	7.08E-05	unchanged
BMB171_RS03615	*gerAA*	spore germination protein KA	27.5	26.7	37.6	34.6	-0.42	0.00122112	unchanged
BMB171_RS03620	*gerAB*	spore germination protein LB	14.4	10.7	23.8	25.2	-0.97	2.00E-06	down
BMB171_RS03625	*gerAC*	spore germination protein LC	23.6	22.7	64.8	54.9	-1.38	5.09E-27	unchanged
BMB171_RS03785	*ylbB*	inosine-5′-monophosphate dehydrogenase	750.9	730.4	1180.1	1354.7	-0.77	2.56E-75	unchanged
BMB171_RS03980	*gerAB*	GerB family spore germination protein	3.4	2.2	6.2	4.4	-0.96	0.02809531	unchanged
BMB171_RS03990	*gerAA*	GerA family spore germination protein	10.5	11.8	16.0	19.6	-0.66	0.00052175	unchanged
BMB171_RS04360	*katX*	catalase	42.6	39.8	50.3	40.4	-0.15	0.11761485	unchanged
BMB171_RS05870	*katX*	catalase	314.3	319.3	236.3	281.6	0.30	5.69E-13	down
BMB171_RS05990	*yjbA*	hypothetical protein	187.5	184.6	784.2	886.8	-2.16	0	down
BMB171_RS07030	*gerW*	hypothetical protein	102.9	91.9	811.3	630.6	-2.91	7.83E-285	unchanged
BMB171_RS07045	*ykuS*	hypothetical protein	5158.9	4909.0	6002.3	6710.9	-0.33	2.22E-47	down

BMB171_RS07585	*yphA*	hypothetical protein	6.1	4.5	14.5	15.3	-1.50	6.37E-05	down
BMB171_RS07590	*seaA*	hypothetical protein	14.1	17.3	39.3	50.0	-1.49	2.14E-17	down
BMB171_RS09100	*yetF*	hypothetical protein	2.2	5.5	20.7	18.1	-2.32	3.32E-11	unchanged
BMB171_RS09415	*arsB*	arsenical-resistance protein ACR3	6.5	4.3	2.8	3.6	0.77	0.09091818	down
BMB171_RS10145	*spoIIP*	stage II sporulation protein P	58.9	58.4	167.4	161.3	-1.49	3.67E-79	down
BMB171_RS10385	*gerW*	hypothetical protein	597.3	630.5	2231.9	1794.1	-1.72	0	down
BMB171_RS13560	*yqzG*	hypothetical protein	25.7	38.2	71.9	60.3	-1.05	2.01E-06	down
BMB171_RS14855	*katX*	catalase	39.0	42.6	88.9	93.9	-1.16	3.78E-36	down
BMB171_RS15390	*gerAC*	spore germination protein QC	1.0	1.0	11.5	9.3	-3.40	1.08E-13	unchanged
BMB171_RS15395	*gerAB*	spore germination protein Qb	0.7	2.2	1.9	3.8	-0.91	0.13860643	unchanged
BMB171_RS15400	*gerAA*	spore germination protein QA	6.2	3.6	9.2	7.7	-0.82	0.00503899	down
BMB171_RS15455	*gerAC*	spore germination protein BC	5.9	6.0	18.3	13.1	-1.41	2.34E-08	unchanged
BMB171_RS15460	*gerAB*	spore germination protein BB	2.8	3.0	6.4	8.2	-1.32	0.00204469	unchanged
BMB171_RS15675	*arsC*	arsenate reductase	32.1	24.6	32.1	35.9	-0.26	0.32122746	unchanged
BMB171_RS15680	*arsB*	arsenical-resistance protein ACR3	3.6	2.6	5.1	5.0	-0.71	0.11434179	unchanged
BMB171_RS16435	*yfkO*	NAD(P)H-dependent flavin reductase	4.3	9.6	6.0	9.1	-0.08	0.86000187	unchanged
BMB171_RS17610	*yfhD*	hypothetical protein	8026.5	7544.9	10106.1	8703.6	-0.28	3.93E-33	unchanged
BMB171_RS17705	*gerAA*	spore germination protein SA	5.3	8.5	10.0	11.9	-0.64	0.00806993	unchanged
BMB171_RS17710	*gerAB*	spore germination protein SB	5.1	4.7	4.3	4.4	0.16	0.70697681	down
BMB171_RS17715	*gerAC*	spore germination protein Sc	4.9	2.7	9.9	8.2	-1.27	0.00016817	unchanged
BMB171_RS17880	*tlP*	small acid-soluble spore protein Tlp	1651.0	1696.9	2065.9	2233.4	-0.36	6.44E-16	unchanged
BMB171_RS17885	*sspN*	acid-soluble spore protein N	5189.7	5178.7	5957.1	6882.3	-0.30	6.45E-23	down
BMB171_RS19195	*ylzA*	hypothetical protein	40.8	31.3	78.4	87.9	-1.21	1.13E-07	down
BMB171_RS19355	*sigG*	sporulation sigma factor SigG	1392.6	1405.5	3404.2	3369.7	-1.28	0	unchanged
BMB171_RS19450	*rsfA*	prespore specific transcriptional activator RsfA	845.5	790.2	1672.5	1572.3	-0.99	6.88E-180	unchanged

BMB171_RS19525	*ylbC*	hypothetical protein	65.0	58.1	80.0	79.3	-0.37	0.00026663	down
BMB171_RS20235	*sigF*	sporulation sigma factor SigF	3093.7	2964.5	12370.6	12571.2	-2.04	0	down
BMB171_RS20240	*spoIIAB*	anti-sigma F factor	1370.5	1336.6	6011.7	6088.9	-2.16	0	down
BMB171_RS20250	*dacF*	D-alanyl-D-alanine carboxypeptidase	617.6	614.7	2229.5	1855.1	-1.74	0	down
BMB171_RS20755	*spoIVB*	stage IV sporulation protein B	581.2	634.6	1773.1	1366.4	-1.38	0	down
BMB171_RS20900	*yqhQ*	hypothetical protein	59.3	53.8	123.2	141.0	-1.22	9.12E-38	unchanged
BMB171_RS21005	*yqhH*	SNF2 family DNA/RNA helicase	44.6	41.1	61.6	63.4	-0.55	3.15E-09	unchanged
BMB171_RS21010	*yqhG*	hypothetical protein	14.9	18.5	30.0	35.2	-0.95	2.39E-06	unchanged
BMB171_RS21555	*gpr*	germination protease	255.7	245.8	506.8	413.7	-0.89	2.71E-93	down
BMB171_RS21825	*yrrS*	hypothetical protein	232.0	185.2	550.1	512.4	-1.36	2.41E-127	down
BMB171_RS21830	*pbpI*	penicillin-binding protein	16.1	17.0	66.2	57.6	-1.91	8.95E-65	down
BMB171_RS21945	*rsfA*	prespore specific transcriptional activator RsfA	149.3	138.7	335.6	322.0	-1.20	4.64E-63	down
BMB171_RS22060	*bofC*	BofC protein	623.3	608.1	1327.5	1496.7	-1.19	2.99E-218	down
BMB171_RS22290	*lonB*	ATP-dependent protease La	84.9	83.3	261.4	314.0	-1.76	1.18E-250	down
BMB171_RS23090	*gerW*	hypothetical protein	1058.2	1023.9	2474.2	1988.6	-1.11	8.99E-234	down
BMB171_RS23095	*ytfI*	hypothetical protein	111.6	123.2	371.9	312.2	-1.55	8.11E-97	down
BMB171_RS23575	*gerAB*	spore germination protein IB	2.7	3.7	9.1	9.8	-1.54	1.28E-05	down
BMB171_RS23850	*mutTA*	MutT/NUDIX family protein phosphohydrolase	150.1	134.0	321.4	339.1	-1.22	7.95E-49	unchanged
BMB171_RS24115	*arsR*	ArsR family transcriptional regulator	10.9	17.6	40.1	21.6	-1.13	0.00196978	down
BMB171_RS24575	*yuiC*	hypothetical protein	325.3	338.4	721.2	705.8	-1.10	1.39E-116	unchanged
BMB171_RS24720	*yutH*	CotS-related protein	64.9	61.1	105.9	89.9	-0.65	1.98E-11	down
BMB171_RS26375	*spoIIQ*	stage II sporulation protein Q	869.6	862.7	2729.9	2777.3	-1.67	0	down
BMB171_RS26615	*spoIIR*	stage II sporulation protein R	39.0	31.0	177.1	109.5	-2.07	8.02E-84	down
BMB171_RS26845	*pbpG*	hypothetical protein	44.2	41.1	95.6	98.6	-1.19	7.05E-59	down

BMB171_RS26895	*rsfA*	prespore specific transcriptional activator	587.0	598.5	1886.4	1853.9	-1.66	0	down
BMB171_RS27410	*yyaC*	hypothetical protein	52.3	51.3	131.2	126.5	-1.32	1.81E-27	unchanged
**The expression level of genes controlled by SigE**									
BMB171_RS00290	*spoVB*	polysaccharides export protein	16.2	18.9	55.7	48.2	-1.57	2.79E-38	down
BMB171_RS01185	*yitE*	hypothetical protein	8.0	11.9	14.1	16.1	-0.59	0.03707687	unchanged
BMB171_RS01265	*yhaX*	HAD superfamily hydrolase	14.9	21.3	22.3	24.2	-0.35	0.10453134	unchanged
BMB171_RS01340	*ydcA*	hypothetical protein	0.0	0.6	0.5	4.2	-2.84	0.02912363	unchanged
BMB171_RS01350	*ydcC*	hypothetical protein	1490.3	1502.7	706.8	727.8	1.06	0	up
BMB171_RS01355	*alrB*	alanine racemase	6008.8	5941.0	1023.6	1061.7	2.52	0	up
BMB171_RS02180	*yugT*	exo-alpha-1,4-glucosidase	3016.5	3013.6	5985.3	6073.6	-1.00	0	unchanged
BMB171_RS02255	*yflB*	hypothetical protein	37.9	22.2	88.2	66.1	-1.39	6.17E-12	down
BMB171_RS02300	*yesK*	hypothetical protein	213.5	183.6	152.8	169.6	0.30	0.01416559	unchanged
BMB171_RS02350	*yloB*	calcium-transporting ATPase	72.6	74.7	124.9	121.8	-0.75	5.61E-45	unchanged
BMB171_RS02485	*yodQ*	acetylornithine deacetylase	4.4	2.7	4.4	7.1	-0.68	0.07265033	unchanged
BMB171_RS02515	*ylaM*	glutaminase	2.8	1.8	3.5	3.0	-0.50	0.39877139	unchanged
BMB171_RS02545	*ywqF*	UDP-N-acetyl-D-mannosamine 6-dehydrogenase	292.5	298.8	7.1	6.7	5.41	0	up
BMB171_RS02655	*yknU*	multidrug ABC transporter	17.5	20.7	35.1	40.9	-0.98	2.95E-15	unchanged
BMB171_RS02840	*yhbB*	hypothetical protein	513.7	485.6	234.3	186.8	1.23	3.43E-137	up
BMB171_RS02870	*yhbH*	hypothetical protein	905.6	910.6	3137.5	3071.9	-1.77	0	down
BMB171_RS03290	*yrbD*	sodium/proton-dependent alanine carrier protein	24.1	20.2	36.7	36.7	-0.73	3.92E-08	unchanged
BMB171_RS03430	*yjmD*	bicyclomycin resistance protein	13.4	13.4	10.9	12.5	0.20	0.44879422	unchanged
BMB171_RS03920	*yhxC*	short chain dehydrogenase	82.7	86.5	194.3	191.7	-1.19	7.92E-50	down
BMB171_RS03970	*yhdB*	hypothetical protein	371.5	400.5	503.3	525.7	-0.41	5.88E-07	unchanged
BMB171_RS04005	*spoVR*	stage V sporulation protein R	543.5	574.1	2076.8	1846.4	-1.82	0	down

BMB171_RS04205	*spoVB*	alkaline phosphatase-like protein	6.1	4.5	1.0	0.0	3.33	0.00012261	up
BMB171_RS04395	*yknU*	multidrug ABC transporter permease/ATP-binding protein	16.4	15.2	33.4	30.3	-1.02	9.90E-12	down
BMB171_RS04400	*yknU*	multidrug ABC transporter permease/ATP-binding protein	29.1	28.0	50.8	49.5	-0.81	9.13E-17	unchanged
BMB171_RS04430	*yheD*	hypothetical protein	209.7	213.0	255.4	253.7	-0.27	9.73E-09	unchanged
BMB171_RS04435	*yheC*	hypothetical protein	36.7	35.9	45.2	49.0	-0.37	0.00583188	unchanged
BMB171_RS04455	*yhcZ*	two-component response regulator comA	10.7	6.3	8.5	10.0	-0.13	0.7513851	unchanged
BMB171_RS04475	*yhaX*	HAD superfamily hydrolase	583.4	550.3	1009.2	1136.1	-0.92	3.06E-177	unchanged
BMB171_RS04495	*yngI*	acyl-CoA synthetase	32.7	31.8	89.6	97.0	-1.53	6.53E-63	down
BMB171_RS04570	*yflJ*	hypothetical protein	507.9	517.0	237.3	315.7	0.91	3.06E-15	unchanged
BMB171_RS05215	*yhaM*	3'-5' exoribonuclease YhaM	79.8	69.5	126.7	132.7	-0.80	1.67E-19	unchanged
BMB171_RS05715	*yhfN*	zinc metalloprotease	27.2	29.5	102.0	98.2	-1.82	1.31E-70	down
BMB171_RS05830	*yisI*	stage 0 sporulation regulatory protein	0.0	1.9	0.0	0.0	NA	NA	unchanged
BMB171_RS05920	*yitE*	hypothetical protein	15.2	13.2	35.8	34.7	-1.32	1.54E-11	down
BMB171_RS05985	*yjaZ*	hypothetical protein	94.2	95.9	53.2	46.1	0.93	9.44E-16	unchanged
BMB171_RS06060	*yjbE*	integral membrane protein	14.3	15.3	50.8	51.8	-1.79	1.06E-19	down
BMB171_RS06180	*spsI*	glucose-1-phosphate thymidylyltransferase	222.5	184.1	1037.3	755.0	-2.16	0	down
BMB171_RS06190	*spsJ*	dTDP-glucose 4,6-dehydratase	356.1	303.1	1205.1	1087.1	-1.81	0	down
BMB171_RS06195	*spsK*	dTDP-4-dehydrorhamnose reductase	292.4	275.5	954.4	870.0	-1.69	0	down
BMB171_RS06215	*yjcA*	integral membrane protein	201.2	186.3	490.1	456.0	-1.29	8.76E-58	down
BMB171_RS06815	*yhfO*	acetyltransferase	16.7	24.9	61.8	59.8	-1.54	2.35E-16	down
BMB171_RS07275	*yhcZ*	two-component response regulator yvqC	81.0	70.6	146.6	127.5	-0.87	3.18E-16	unchanged
BMB171_RS07420	*sodF*	superoxide dismutase	345.9	347.6	836.6	891.7	-1.31	1.72E-268	down
BMB171_RS07430	*spmA*	spore maturation protein A	31.7	17.3	92.1	78.3	-1.82	3.04E-29	down
BMB171_RS07435	*spmB*	spore maturation protein B	19.6	24.6	101.3	104.3	-2.21	1.42E-39	down

BMB171_RS07445	*stoA*	thiol-disulfide oxidoreductase	7.1	7.9	18.1	11.9	-1.02	0.00657721	unchanged
BMB171_RS07515	*ypbG*	phosphoesterase	37.3	31.9	76.5	47.2	-0.87	1.10E-09	unchanged
BMB171_RS07625	*spoIVA*	stage IV sporulation protein A	1885.5	1922.0	3311.3	3828.0	-0.90	0	unchanged
BMB171_RS07675	*ypiA*	TPR repeat-containing protein	118.5	145.4	218.1	238.5	-0.78	1.48E-42	unchanged
BMB171_RS07715	*ypjB*	hypothetical protein	10.8	11.3	77.5	55.7	-2.61	1.31E-46	down
BMB171_RS08155	*ywcA*	sodium/proline symporter	19.6	17.3	77.2	77.7	-2.07	5.10E-79	down
BMB171_RS08160	*yodT*	aminotransferase	464.6	457.8	1930.6	1789.3	-2.02	0	down
BMB171_RS08845	*stoA*	Thiol:disulfide interchange protein	1.3	2.5	5.3	7.5	-1.71	0.00738585	unchanged
BMB171_RS08900	*yhcZ*	two-component response regulator	0.6	0.0	6.5	2.7	-4.08	5.87E-05	down
BMB171_RS09480	*ydbI*	hypothetical protein	64.5	62.7	59.8	72.7	-0.05	0.6890769	unchanged
BMB171_RS09490	*yobN*	amine oxidase	11.9	7.9	23.0	27.5	-1.35	5.36E-14	down
BMB171_RS09570	*yknU*	transport ATP-binding protein CydC	11.4	10.4	20.0	16.3	-0.75	2.01E-05	unchanged
BMB171_RS09645	*yknU*	multidrug ABC transporter	31.2	33.6	31.6	32.4	0.02	0.87430383	unchanged
BMB171_RS09895	*ytvB*	hypothetical protein	126.0	114.6	171.6	142.3	-0.40	0.00183829	unchanged
BMB171_RS09930	*ykvI*	hypothetical protein	90.6	86.9	254.4	349.3	-1.75	1.69E-161	down
BMB171_RS09960	*yobN*	amine oxidase	8.9	11.7	21.2	28.4	-1.24	2.43E-12	down
BMB171_RS10055	*spoVB*	polysaccharides/teichoic acids export protein	4.6	7.0	27.2	35.6	-2.42	8.38E-35	down
BMB171_RS10145	*spoIIP*	stage II sporulation protein P	58.9	58.4	167.4	161.3	-1.49	3.67E-79	down
BMB171_RS10195	*yhaX*	HAD superfamily hydrolase	1.0	4.0	3.0	3.1	-0.28	0.67075129	unchanged
BMB171_RS10210	*ymxH*	alanine racemase	92.2	83.0	118.4	127.8	-0.49	3.32E-10	unchanged
BMB171_RS10265	*yhfO*	acetyltransferase	9.9	6.4	16.4	17.1	-1.05	0.00489686	unchanged
BMB171_RS10785	*yxjC*	D-beta-hydroxybutyrate permease	8.6	11.1	45.5	34.1	-2.02	8.17E-32	down
BMB171_RS10805	*yhfO*	acetyltransferase	100.2	86.6	111.9	83.2	-0.08	0.57222259	unchanged
BMB171_RS10875	*spoVD*	MecA protein	36.3	36.5	51.9	51.8	-0.51	3.76E-08	unchanged
BMB171_RS10990	*yhcY*	two-component sensor protein YhcY	4.0	4.9	12.9	9.3	-1.33	1.55E-05	down
BMB171_RS10995	*yhcZ*	two-component response regulator YhcZ	4.5	4.8	5.6	4.2	-0.10	0.87302439	unchanged

BMB171_RS11135	*yodT*	hypothetical protein	42.8	43.3	130.1	104.1	-1.46	1.33E-62	down
BMB171_RS11140	*scoA*	3-keto-6-acetamidohexanoate cleavage protein	91.4	110.7	286.7	260.6	-1.44	7.64E-76	down
BMB171_RS11145	*scoB*	3-keto-6-acetamidohexanoate cleavage protein	163.6	145.2	524.7	423.6	-1.63	6.13E-150	down
BMB171_RS11150	*yodQ*	acetylornithine deacetylase	255.3	260.4	1243.0	840.8	-2.04	0	down
BMB171_RS11155	*yodP*	beta-lysine acetyltransferase	329.0	331.7	1198.0	985.8	-1.74	0	down
BMB171_RS11170	*yokU*	hypothetical protein	1251.9	1211.8	1157.3	1205.1	0.06	0.22450793	unchanged
BMB171_RS11175	*yozE*	hypothetical protein	7.7	14.3	10.8	7.2	0.29	0.68028671	unchanged
BMB171_RS11180	*yodN*	hypothetical protein	556.4	535.8	1356.1	1464.1	-1.37	0	down
BMB171_RS11190	*yozD*	hypothetical protein	8952.7	8778.7	18731.8	21397.3	-1.17	0	down
BMB171_RS11345	*yqiQ prpB*	methylisocitrate lyase	430.3	413.9	613.9	663.3	-0.59	1.07E-52	unchanged
BMB171_RS11735	*yknU*	multidrug ABC transporter permease/ATP-binding protein	28.4	28.4	23.2	22.1	0.32	0.016321	unchanged
BMB171_RS11740	*yknU*	multidrug ABC transporter permease/ATP-binding protein	42.4	42.3	32.2	36.2	0.31	0.00346977	unchanged
BMB171_RS12255	*yhcZ*	two-component protein kinase	43.0	58.4	33.9	39.3	0.49	0.0003553	unchanged
BMB171_RS12265	*spoVD*	cell elongation specific D,D-transpeptidase	9.5	9.2	25.0	16.2	-1.17	1.87E-11	down
BMB171_RS12330	*yngJ*	short chain acyl-CoA dehydrogenase	291.7	288.7	662.3	592.9	-1.12	2.29E-191	down
BMB171_RS12335	*yngH*	acetyl-CoA carboxylase biotin carboxylase subunit	490.3	514.3	1274.6	1244.6	-1.33	0	down
BMB171_RS12340	*yngHB*	acetyl-CoA carboxylase biotin carboxyl carrier protein subunit	1057.7	1122.4	2052.5	2451.3	-1.03	3.89E-113	down
BMB171_RS12345	*yngG*	hydroxymethylglutaryl-CoA lyase	195.9	206.6	368.8	390.3	-0.91	6.80E-66	unchanged
BMB171_RS12350	*yngF*	enoyl-CoA hydratase	215.2	238.0	439.0	463.0	-0.99	2.27E-77	unchanged

BMB171_RS12355	*yngE*	propionyl-CoA carboxylase beta chain	252.5	265.4	437.6	506.9	-0.86	1.30E-123	unchanged
BMB171_RS13050	*yhaX*	HAD superfamily hydrolase	7.0	7.7	22.2	31.7	-1.86	6.40E-14	down
BMB171_RS13120	*spoVD*	division specific D,D-transpeptidase/cell division protein FtsI	44.3	33.7	183.3	168.5	-2.19	8.99E-249	down
BMB171_RS13810	*ywnH*	phosphinothricin N-acetyltransferase	55.8	38.2	58.8	64.8	-0.40	0.01920103	unchanged
BMB171_RS14345	*ydbI*	hypothetical protein	22.9	27.2	29.4	37.6	-0.40	0.01212472	unchanged
BMB171_RS14470	*yjbE*	integral membrane protein	132.6	124.8	238.6	242.2	-0.90	4.01E-31	unchanged
BMB171_RS15300	*yesJ*	acetyltransferase	1.4	2.7	1.7	2.7	-0.06	0.94960506	unchanged
BMB171_RS15310	*yhjR*	hypothetical protein	518.4	531.2	211.3	160.0	1.48	2.44E-94	up
BMB171_RS15360	*yjmD*	glutathione-dependent formaldehyde dehydrogenase	156.6	138.9	284.9	261.1	-0.89	1.22E-57	unchanged
BMB171_RS15480	*ylaM*	glutaminase	4.5	4.6	5.7	4.9	-0.24	0.59026013	unchanged
BMB171_RS15525	*ydjP*	arylesterase	1.8	0.8	0.7	0.8	0.75	0.47664874	unchanged
BMB171_RS15870	*yetO*	NADPH-cytochrome P450 reductase	121.3	117.7	373.2	332.2	-1.57	0	down
BMB171_RS16270	*ydjP*	3-oxoadipate enol-lactonase	584.6	626.1	833.6	989.8	-0.58	9.57E-71	unchanged
BMB171_RS16460	*spoIIIAH*	hypothetical protein	697.4	707.9	4117.2	3371.2	-2.42	0	down
BMB171_RS16545	*ydbI*	hypothetical protein	19.9	18.4	10.3	14.2	0.66	0.00198645	unchanged
BMB171_RS16945	*ydhD*	peptidoglycan N-acetylglucosamine deacetylase	3.1	2.6	2.6	2.1	0.28	0.39124294	unchanged
BMB171_RS17170	*yeaA*	hypothetical protein	13.5	6.6	26.7	24.6	-1.37	2.39E-10	down
BMB171_RS17185	*yjfA*	hypothetical protein	62.4	52.8	480.5	311.4	-2.81	3.00E-156	down
BMB171_RS17190	*yjfA*	hypothetical protein	36.9	32.2	461.5	227.5	-3.36	4.25E-199	down
BMB171_RS17375	*yfnD*	glycosyltransferase	444.0	423.6	18.0	15.9	4.67	0	up
BMB171_RS17605	*yxjF*	3-ketoacyl-(acyl-carrier-protein) reductase	5.5	7.0	13.6	9.8	-0.92	0.00925127	unchanged
BMB171_RS17615	*sqhC*	squalene--hopene cyclase	58.3	61.4	80.3	94.9	-0.54	2.97E-13	unchanged
BMB171_RS17875	*ydhD*	spore peptidoglycan hydrolase	278.7	284.8	297.4	320.0	-0.13	0.00520612	unchanged

BMB171_RS17900	*stoA*	Thiol:disulfide interchange protein	89.6	87.8	343.3	221.2	-1.69	5.79E-83	down
BMB171_RS18030	*yitE*	hypothetical protein	0.5	0.0	0.7	0.4	-1.42	0.41803111	unchanged
BMB171_RS18220	*yknU*	multidrug ABC transporter permease/ATP-binding protein	20.4	15.6	35.5	37.3	-1.02	1.01E-15	down
BMB171_RS18225	*yknU*	multidrug ABC transporter permease/ATP-binding protein	1.1	3.1	6.7	6.1	-1.59	2.66E-06	down
BMB171_RS18385	*spoVK*	cell division protein FtsH	263.6	269.9	412.9	349.2	-0.52	3.17E-27	unchanged
BMB171_RS18850	*ylmC*	hypothetical protein	59.0	84.6	267.4	131.9	-1.50	1.01E-22	down
BMB171_RS19130	*yloS*	thiamin pyrophosphokinase	2.9	7.5	13.4	13.5	-1.34	0.00036251	down
BMB171_RS19205	*yloB*	calcium-transporting ATPase	133.6	133.4	503.7	436.9	-1.82	0	down
BMB171_RS19395	*spoVE*	stage V sporulation protein E	92.9	81.9	162.1	196.0	-1.03	1.10E-45	down
BMB171_RS19415	*spoVD*	stage V sporulation protein D/sporulation specific D,D-transpeptidase	100.6	99.6	359.1	351.5	-1.83	0	down
BMB171_RS19420	*spoVD*	division specific D,D-transpeptidase/cell division protein FtsI	46.9	53.5	90.5	92.0	-0.86	1.29E-33	unchanged
BMB171_RS19475	*ylbK*	serine protease	13.1	12.6	40.7	33.2	-1.54	7.33E-14	down
BMB171_RS19480	*ylbJ*	hypothetical protein	4.2	6.7	12.5	15.2	-1.32	6.24E-07	down
BMB171_RS19575	*yngH*	pyruvate carboxylase	409.1	417.3	243.0	262.6	0.71	6.88E-177	unchanged
BMB171_RS19595	*ylaK*	PhoH family protein	756.9	772.7	2077.1	1811.5	-1.35	0	down
BMB171_RS19720	*yhaX*	HAD superfamily hydrolase	7.2	4.4	12.6	13.0	-1.15	0.00076446	down
BMB171_RS20010	*yxjF*	3-hydroxybutyrate dehydrogenase	22.6	16.6	57.3	65.8	-1.65	2.70E-24	down
BMB171_RS20335	*spoIIM*	stage II sporulation protein M	7.2	3.9	33.0	23.5	-2.38	7.64E-15	down
BMB171_RS20470	*yodT*	adenosylmethionine--8-amino-7-oxononanoate transaminase	2.1	1.2	2.3	3.7	-0.84	0.10616255	unchanged
BMB171_RS20810	*yngH*	acetyl-CoA carboxylase biotin carboxylase subunit	720.7	704.6	917.9	1047.8	-0.46	2.75E-74	unchanged

BMB171_RS20825	*spoIIIAH*	stage III sporulation protein AH	1060.5	1051.4	4394.2	3832.8	-1.97	0	down
BMB171_RS20835	*spoIIIAF*	stage III sporulation protein AF	404.9	397.7	2686.6	2281.3	-2.64	0	down
BMB171_RS20840	*spoIIIAE*	stage III sporulation protein AE	17.7	13.8	48.9	46.1	-1.60	1.12E-26	down
BMB171_RS20845	*spoIIIAD*	stage III sporulation protein AD	29.3	18.5	95.3	96.8	-2.02	5.02E-18	down
BMB171_RS20855	*spoIIIAB*	stage III sporulation protein SpoAB	69.5	61.0	263.3	192.4	-1.83	4.78E-66	down
BMB171_RS20860	*spoIIIAA*	stage III sporulation protein AA	70.9	63.4	251.9	178.9	-1.70	6.33E-102	down
BMB171_RS20865	*yqhV*	hypothetical protein	96.2	50.0	201.2	115.4	-1.16	5.44E-14	down
BMB171_RS21285	*spsI*	phosphoglucomutase	51.6	45.3	256.0	247.6	-2.38	0	down
BMB171_RS21325	*sodF*	superoxide dismutase	323.5	348.9	477.1	481.5	-0.51	6.00E-21	unchanged
BMB171_RS21475	*yqfD*	stage IV sporulation protein	45.5	40.7	163.5	151.5	-1.88	3.89E-109	down
BMB171_RS21480	*yqfC*	hypothetical protein	53.1	58.5	137.7	155.5	-1.38	5.57E-17	down
BMB171_RS21895	*ydbI*	hypothetical protein	37.6	38.0	88.6	85.3	-1.20	1.01E-28	down
BMB171_RS22010	*spoVB*	stage V sporulation protein B	8.1	7.3	7.6	9.8	-0.16	0.54976525	unchanged
BMB171_RS22015	*yrbG*	hypothetical protein	8.0	4.8	11.4	11.2	-0.84	0.02911554	unchanged
BMB171_RS22175	*spoIVFA*	stage IV sporulation protein FA	27.8	32.7	96.9	118.2	-1.81	1.53E-44	down
BMB171_RS22230	*ysxE*	CotS-related protein	2521.1	2568.4	4211.3	3389.0	-0.59	0	unchanged
BMB171_RS22275	*ylxY*	osmotically inducible protein C	228.1	264.7	280.7	363.7	-0.37	4.36E-06	unchanged
BMB171_RS22320	*yvbH*	hypothetical protein	144.4	138.7	136.5	171.2	-0.11	0.26753366	unchanged
BMB171_RS22475	*yslB*	hypothetical protein	9.1	13.0	15.1	11.5	-0.28	0.49761372	unchanged
BMB171_RS22500	*yngF*	enoyl-CoA hydratase	853.4	848.4	1824.3	1763.1	-1.08	0	down
BMB171_RS22510	*yngI*	acyl-CoA synthase	241.5	222.2	1086.4	1058.1	-2.21	0	down
BMB171_RS22725	*sodF*	multidrug resistance protein B	25.6	32.3	11.3	17.9	1.02	5.13E-13	up
BMB171_RS22770	*ytxC*	hypothetical protein	15.2	16.0	79.8	76.2	-2.32	9.84E-53	down
BMB171_RS22810	*ytaF*	integral membrane protein	700.1	695.9	439.8	482.0	0.60	1.05E-41	unchanged
BMB171_RS22870	*ydbI*	hypothetical protein	10.6	19.4	24.3	30.0	-0.83	5.51E-06	unchanged
BMB171_RS22915	*ytrH*	hypothetical protein	5.5	20.3	75.9	91.9	-2.66	7.78E-26	down

BMB171_RS23440	*spoVB*	polysaccharides/teichoic acids export protein	6.3	7.3	9.8	11.1	-0.61	0.01010223	unchanged
BMB171_RS23470	*yteV*	hypothetical protein	862.7	779.9	156.7	166.5	2.35	1.42E-114	up
BMB171_RS23485	*yjmF*	glucose-1-dehydrogenase	201.8	187.3	390.3	355.5	-0.95	5.31E-60	unchanged
BMB171_RS23795	*ydjP*	non-heme chloroperoxidase	346.3	368.5	526.9	433.1	-0.43	5.73E-21	unchanged
BMB171_RS24220	*ydjP*	menaquinone biosynthesis related protein	16.0	14.0	40.0	43.4	-1.48	6.64E-15	down
BMB171_RS24660	*yutJ*	NADH dehydrogenase	71.1	67.1	63.5	66.9	0.09	0.44738684	unchanged
BMB171_RS24720	*yutH*	CotS-related protein	64.9	61.1	105.9	89.9	-0.65	1.98E-11	unchanged
BMB171_RS25030	*yycO*	YycO protein	3.4	5.9	20.3	16.9	-1.99	6.20E-10	down
BMB171_RS25035	*yycP*	hypothetical protein	0.0	1.1	0.2	0.3	1.16	0.39549987	unchanged
BMB171_RS25070	*yitE*	hypothetical protein	1.7	2.7	2.3	3.5	-0.38	0.55756523	unchanged
BMB171_RS25770	*spsJ*	LysR family transcriptional regulator	8.3	5.4	10.9	12.4	-0.77	0.01502977	unchanged
BMB171_RS25795	*yknU*	multidrug ABC transporter	2.6	2.4	5.0	4.8	-0.98	0.00688133	unchanged
BMB171_RS25925	*ywqF*	UDP-glucose 6-dehydrogenase	12.9	20.2	47.5	40.5	-1.41	2.69E-23	down
BMB171_RS26275	*spsK*	dTDP-4-dehydrorhamnose 3,5-epimerase	28.7	19.3	43.2	53.1	-1.00	3.39E-10	down
BMB171_RS26285	*spsI*	polysaccharides/teichoic acids export protein	8.9	11.3	18.5	17.3	-0.82	0.00129299	unchanged
BMB171_RS26305	*ywqF*	nucleotide sugar dehydrogenase subfamily protein	4.6	6.5	9.9	11.0	-0.91	0.00162314	unchanged
BMB171_RS26330	*spsI*	UTP--glucose-1-phosphate uridylyltransferase	1.7	1.6	1.7	0.8	0.36	0.68128151	unchanged
BMB171_RS26365	*spoIIID*	stage III sporulation protein D	8511.4	8477.6	8610.1	10744.1	-0.18	8.45E-25	unchanged
BMB171_RS26395	*spoIID*	stage II sporulation protein D	692.3	721.5	1413.2	1081.0	-0.83	3.29E-208	unchanged
BMB171_RS26685	*yngJ*	short chain acyl-CoA dehydrogenase	345.4	338.2	850.6	745.1	-1.23	2.27E-281	down
BMB171_RS26690	*yngJ*	short chain acyl-CoA dehydrogenase	171.1	160.8	390.7	302.2	-1.08	3.39E-99	down
BMB171_RS26740	*yhcZ*	two-component response regulator YocG	11.1	15.4	26.5	23.5	-0.91	0.00053328	unchanged
BMB171_RS26950	*yhaX*	HAD superfamily hydrolase	17.9	10.2	8.8	11.5	0.47	0.11279583	unchanged
BMB171_RS27025	*yhcZ*	two-component response regulator YhcZ	6.9	8.0	14.1	19.8	-1.17	0.00033077	down

BMB171_RS27155	*ydbI*	hypothetical protein	63.3	54.0	61.1	79.9	-0.25	0.0201503	unchanged
BMB171_RS27260	*yknU*	multidrug ABC transporter	0.6	1.2	1.3	1.2	-0.42	0.48845048	unchanged

Table S5. Analysis of the differential expression genes implicated in KEGG term pathways

Gene ID	Length	Function	RPKM				log2(fold change) (Δ3*hfq* *vs* BMB171)	*q*-vaule	Change (Δ3*hfq* *vs* BMB171)
			Δ3*hfq*-1	Δ3*hfq*-2	171-1	171-2			
**Amino acid metabolism**									
**Alanine, aspartate and glutamate metabolism (btb00250)**									
BMB171_RS00950	1803	D-fructose-6-phosphate amidotransferase	345.9	344.7	132.5	170.2	1.20	1.93E-185	up
BMB171_RS01900	1452	succinate-semialdehyde dehydrogenase	304.7	285.2	942.4	878.6	-1.63	0	down
BMB171_RS07815	1188	aspartate aminotransferase	471.2	468.9	143.3	163.8	1.62	2.18E-260	up
BMB171_RS08980	984	asparagine synthetase AsnA	9.8	10.1	21.3	19.7	-1.04	6.22E-06	down
BMB171_RS09960	1464	amine oxidase	8.9	11.7	21.2	28.4	-1.24	2.43E-12	down
BMB171_RS10930	1902	asparagine synthetase	73.2	72.3	145.0	154.9	-1.04	1.29E-67	down
BMB171_RS19270	3219	carbamoyl phosphate synthase large subunit	67.2	66.8	165.4	159.2	-1.28	2.11E-171	down
BMB171_RS19275	1098	carbamoyl phosphate synthase small subunit	64.6	65.8	149.4	151.7	-1.21	3.24E-50	down
BMB171_RS19285	915	aspartate carbamoyltransferase	22.3	18.0	60.4	46.9	-1.43	2.25E-20	down
**Glycine, serine and threonine metabolism (btb00260)**									
BMB171_RS04995	534	CDP-diacylglycerol-serine O-phosphatidyltransferase	9.0	13.5	32.1	20.8	-1.24	1.28E-05	down
BMB171_RS06305	1194	tryptophan synthase subunit beta	11.5	9.8	29.7	27.3	-1.43	2.35E-14	down
BMB171_RS06310	777	tryptophan synthase subunit alpha	11.0	15.0	55.9	48.1	-2.00	2.19E-26	down

BMB171_RS07160	702	CDP-diacylglycerol--serine O-phosphatidyltransferase	82.0	75.0	150.3	171.2	-1.03	5.16E-27	down
BMB171_RS08680	576	phosphoglycerate mutase	10.3	9.0	25.1	25.9	-1.41	1.07E-06	down
BMB171_RS09155	1263	threonine dehydratase	37.1	36.2	97.1	74.6	-1.24	2.39E-35	down
BMB171_RS09700	1059	threonine synthase	269.4	293.2	578.6	600.4	-1.06	4.70E-152	down
BMB171_RS09705	894	homoserine kinase	173.7	172.7	484.0	439.6	-1.42	1.79E-159	down
BMB171_RS11695	1044	aspartate-semialdehyde dehydrogenase	133.8	124.5	50.7	43.1	1.45	2.78E-54	up
BMB171_RS13665	1380	dihydrolipoamide dehydrogenase	272.4	267.6	545.5	583.2	-1.06	1.92E-188	down
BMB171_RS13940	1176	sarcosine oxidase subunit beta	1.6	0.9	6.3	2.1	-1.84	0.00058381	down
BMB171_RS15990	1254	phosphoserine aminotransferase	15.7	11.2	25.0	30.1	-1.03	4.43E-09	down
BMB171_RS18830	1233	aspartate kinase I	3169.2	3100.0	1388.1	1401.5	1.17	0	up
BMB171_RS18835	1047	aspartate-semialdehyde dehydrogenase	2954.5	2913.4	1380.4	1385.2	1.09	0	up
BMB171_RS21800	1134	cystathionine beta-lyase	232.6	215.9	82.1	103.5	1.28	2.82E-84	up
BMB171_RS26990	1296	homoserine dehydrogenase	178.9	181.0	616.0	444.4	-1.58	0	down
**Cysteine and methionine metabolism (btb00270)**									
BMB171_RS02000	1047	translation initiation factor IF-2B	18.4	13.5	40.4	36.7	-1.29	1.68E-14	down
BMB171_RS07815	1188	aspartate aminotransferase	471.2	468.9	143.3	163.8	1.62	2.18E-260	up
BMB171_RS09065	918	cysteine synthase A	57.1	56.2	116.9	121.1	-1.07	6.59E-28	down
BMB171_RS09485	945	L-lactate dehydrogenase	143.1	163.4	311.2	374.1	-1.15	3.41E-89	down
BMB171_RS09960	1464	amine oxidase	8.9	11.7	21.2	28.4	-1.24	2.43E-12	down
BMB171_RS11695	1044	aspartate-semialdehyde dehydrogenase	133.8	124.5	50.7	43.1	1.45	2.78E-54	up
BMB171_RS15900	996	D-cysteine desulfhydrase	16.4	16.6	42.4	46.6	-1.43	3.33E-18	down
BMB171_RS18830	1233	aspartate kinase I	3169.2	3100.0	1388.1	1401.5	1.17	0	up

BMB171_RS18835	1047	aspartate-semialdehyde dehydrogenase	2954.5	2913.4	1380.4	1385.2	1.09	0	up
BMB171_RS19865	2289	5-methyltetrahydropteroyltriglutamate--homocysteine methyltransferase	52.9	58.1	138.9	155.5	-1.40	3.00E-126	down
BMB171_RS21225	1833	bifunctional homocysteine S-methyltransferase/5,10-methylenetetrahydrofolate reductase	81.5	76.5	41.4	30.8	1.11	7.68E-39	up
BMB171_RS21230	1113	cystathionine gamma-synthase	74.1	74.8	27.4	24.6	1.51	5.09E-36	up
BMB171_RS21235	1164	cystathionine beta-lyase	60.6	65.0	21.8	27.4	1.37	1.29E-27	up
BMB171_RS21800	1134	cystathionine beta-lyase	232.6	215.9	82.1	103.5	1.28	2.82E-84	up
BMB171_RS21815	639	dimethyladenosine transferase	41.9	45.7	100.4	90.0	-1.12	2.18E-17	down
BMB171_RS23160	1176	methionine gamma-lyase	60.0	60.8	24.9	23.0	1.33	7.45E-26	up
BMB171_RS23560	999	homoserine O-acetyltransferase	23.1	14.8	60.4	52.3	-1.59	7.18E-27	down
BMB171_RS24495	1152	aspartate aminotransferase	2.6	1.8	6.7	6.0	-1.55	0.00023967	down
BMB171_RS25980	1110	spermidine synthase	77.3	73.1	0.0	1.9	6.45	4.27E-116	up
BMB171_RS26825	828	spermidine synthase	62.8	68.9	137.4	151.8	-1.13	6.05E-33	down
BMB171_RS26990	1296	homoserine dehydrogenase	178.9	181.0	616.0	444.4	-1.58	0	down
BMB171_RS26995	906	homoserine O-succinyltransferase	248.5	233.4	608.1	470.9	-1.18	1.99E-142	down
BMB171_RS27000	1299	O-acetylhomoserine aminocarboxypropyltransferase	173.6	193.5	602.0	500.1	-1.59	0	down
**Valine, leucine and isoleucine degradation (btb00280)**									
BMB171_RS06495	1368	aldehyde dehydrogenase	2.2	6.8	15.3	17.7	-1.85	9.77E-14	down
BMB171_RS07060	897	branched-chain amino acid aminotransferase	151.6	154.9	494.2	489.5	-1.68	3.90E-217	down
BMB171_RS09430	420	methylmalonyl CoA epimerase	7.1	7.4	26.0	21.4	-1.73	2.84E-06	down

BMB171_RS09960	1464	amine oxidase	8.9	11.7	21.2	28.4	-1.24	2.43E-12	down
BMB171_RS11355	891	3-hydroxyisobutyrate dehydrogenase	331.9	352.8	961.3	889.6	-1.44	0	down
BMB171_RS11360	1461	methylmalonate-semialdehyde dehydrogenase	666.7	663.3	1369.6	1550.2	-1.13	0	down
BMB171_RS11370	1056	enoyl-CoA hydratase	9.8	5.9	15.7	21.3	-1.23	4.22E-07	down
BMB171_RS13665	1380	dihydrolipoamide dehydrogenase	272.4	267.6	545.5	583.2	-1.06	1.92E-188	down
BMB171_RS13920	1485	aldehyde dehydrogenase	20.3	22.7	42.2	48.7	-1.07	1.16E-17	down
BMB171_RS26700	1182	acetyl-CoA acetyltransferase	443.8	422.2	1363.3	942.2	-1.43	0	down
**Valine, leucine and isoleucine biosynthesis (btb00290)**									
BMB171_RS07060	897	branched-chain amino acid aminotransferase	151.6	154.9	494.2	489.5	-1.68	3.90E-217	down
BMB171_RS07065	1701	acetolactate synthase 3 catalytic subunit	236.3	237.8	607.4	549.7	-1.29	0	down
BMB171_RS07075	1011	ketol-acid reductoisomerase	505.2	539.8	1454.7	1277.3	-1.39	0	down
BMB171_RS07080	1503	2-isopropylmalate synthase	407.1	389.4	1326.8	1006.4	-1.57	0	down
BMB171_RS07085	1065	3-isopropylmalate dehydrogenase	314.3	321.0	1012.7	771.0	-1.50	0	down
BMB171_RS07090	1395	isopropylmalate isomerase large subunit	115.0	143.5	420.3	360.4	-1.60	4.92E-250	down
BMB171_RS07095	582	isopropylmalate isomerase small subunit	106.0	95.1	387.5	346.3	-1.88	2.88E-123	down
BMB171_RS09135	1716	acetolactate synthase 3 catalytic subunit	23.2	20.4	48.6	51.5	-1.20	2.31E-26	down
BMB171_RS09150	1674	dihydroxy-acid dehydratase	34.0	24.7	61.3	55.0	-1.00	9.19E-23	down
BMB171_RS09155	1263	threonine dehydratase	37.1	36.2	97.1	74.6	-1.24	2.39E-35	down
**Lysine biosynthesis (btb00300)**									
BMB171_RS02225	3582	peptide synthetase	0.7	1.2	3.1	3.4	-1.76	3.68E-07	down

BMB171_RS11695	1044	aspartate-semialdehyde dehydrogenase	133.8	124.5	50.7	43.1	1.45	2.78E-54	up
BMB171_RS13925	897	dihydrodipicolinate synthase	6.6	8.1	16.1	20.1	-1.29	1.88E-06	down
BMB171_RS18620	1434	GntR family transcriptional regulator	4.9	9.1	16.1	20.5	-1.35	2.05E-10	down
BMB171_RS18825	879	dihydrodipicolinate synthase	4006.0	4069.5	1488.2	1531.0	1.42	0	up
BMB171_RS18830	1233	aspartate kinase I	3169.2	3100.0	1388.1	1401.5	1.17	0	up
BMB171_RS18835	1047	aspartate-semialdehyde dehydrogenase	2954.5	2913.4	1380.4	1385.2	1.09	0	up
BMB171_RS19410	1476	UDP-N-acetylmuramoylalanyl-D-glutamate--2, 6-diaminopimelate ligase	34.5	29.8	80.9	68.0	-1.23	6.72E-35	down
BMB171_RS19745	1131	N-acetyldiaminopimelate deacetylase	16.8	7.0	24.6	24.8	-1.08	4.17E-08	down
BMB171_RS24650	867	diaminopimelate epimerase	833.4	843.3	297.6	317.9	1.45	1.78E-287	up
**Lysine degradation (btb00310)**									
BMB171_RS01900	1452	succinate-semialdehyde dehydrogenase	304.7	285.2	942.4	878.6	-1.63	0	down
BMB171_RS02225	3582	peptide synthetase	0.7	1.2	3.1	3.4	-1.76	3.68E-07	down
BMB171_RS06495	1368	aldehyde dehydrogenase	2.2	6.8	15.3	17.7	-1.85	9.77E-14	down
BMB171_RS10955	876	D-amino acid aminotransferase	55.1	45.1	113.0	114.6	-1.19	4.43E-30	down
BMB171_RS11140	699	3-keto-6-acetamidohexanoate cleavage protein	91.4	110.7	286.7	260.6	-1.44	7.64E-76	down
BMB171_RS13920	1485	aldehyde dehydrogenase	20.3	22.7	42.2	48.7	-1.07	1.16E-17	down
BMB171_RS26100	921	D-amino acid aminotransferase	25.8	23.1	66.7	59.0	-1.37	2.68E-22	down
BMB171_RS26700	1182	acetyl-CoA acetyltransferase	443.8	422.2	1363.3	942.2	-1.43	0	down
**Arginie and proline metabolism (btb00330)**									
BMB171_RS01105	804	pyrroline-5-carboxylate reductase	6.5	6.0	18.4	20.2	-1.63	2.02E-08	down

BMB171_RS06495	1368	aldehyde dehydrogenase	2.2	6.8	15.3	17.7	-1.85	9.77E-14	down
BMB171_RS07815	1188	aspartate aminotransferase	471.2	468.9	143.3	163.8	1.62	2.18E-260	up
BMB171_RS10765	876	proline iminopeptidase	423.6	459.7	1159.3	875.6	-1.22	1.77E-272	down
BMB171_RS10955	876	D-amino acid aminotransferase	55.1	45.1	113.0	114.6	-1.19	4.43E-30	down
BMB171_RS11150	1269	acetylornithine deacetylase	255.3	260.4	1243.0	840.8	-2.04	0	down
BMB171_RS13920	1485	aldehyde dehydrogenase	20.3	22.7	42.2	48.7	-1.07	1.16E-17	down
BMB171_RS20510	951	ornithine carbamoyltransferase	24.6	26.0	72.1	71.4	-1.50	1.51E-29	down
BMB171_RS20515	1161	acetylornithine aminotransferase	14.1	15.7	45.1	39.1	-1.50	1.21E-21	down
BMB171_RS20520	768	acetylglutamate kinase	3.9	3.1	18.5	13.1	-2.20	9.13E-10	down
BMB171_RS20525	1224	bifunctional ornithine acetyltransferase/N-acetylglutamate synthase	2.4	3.4	10.1	7.4	-1.60	4.04E-06	down
BMB171_RS20530	1038	aspartate-semialdehyde dehydrogenase	2.5	2.3	10.0	5.3	-1.70	3.32E-05	down
BMB171_RS21130	747	prolyl 4-hydroxylase subunit alpha	7.5	11.0	47.5	32.5	-2.12	1.83E-21	down
BMB171_RS25980	1110	spermidine synthase	77.3	73.1	0.0	1.9	6.45	4.27E-116	up
BMB171_RS26100	921	D-amino acid aminotransferase	25.8	23.1	66.7	59.0	-1.37	2.68E-22	down
BMB171_RS26820	873	agmatinase	83.4	97.7	235.0	209.0	-1.30	7.47E-66	down
BMB171_RS26825	828	spermidine synthase	62.8	68.9	137.4	151.8	-1.13	6.05E-33	down
**Histidine metabolism (btb00340)**									
BMB171_RS06145	753	methyltransferase	34.5	42.0	111.9	99.8	-1.47	2.30E-33	down
BMB171_RS06495	1368	aldehyde dehydrogenase	2.2	6.8	15.3	17.7	-1.85	9.77E-14	down
BMB171_RS07100	1263	ATP phosphoribosyltransferase regulatory subunit	33.8	25.9	85.6	75.9	-1.45	1.17E-41	down
BMB171_RS07105	636	ATP phosphoribosyltransferase catalytic subunit	22.2	26.5	89.2	67.5	-1.70	3.36E-26	down

BMB171_RS07110	1290	histidinol dehydrogenase	47.8	38.4	128.3	96.4	-1.40	3.03E-56	down
BMB171_RS07115	585	imidazoleglycerol-phosphate dehydratase	50.2	51.1	193.5	139.7	-1.74	1.13E-51	down
BMB171_RS07125	720	1-(5-phosphoribosyl)-5-[(5-phosphoribosylamino)methylideneamino] imidazole-4-carboxamide isomerase	43.3	35.8	129.7	86.1	-1.48	3.93E-33	down
BMB171_RS07130	759	imidazole glycerol phosphate synthase	28.9	44.4	160.8	104.9	-1.87	2.51E-59	down
BMB171_RS07135	306	phosphoribosyl-AMP cyclohydrolase	19.4	27.0	218.3	75.8	-2.71	9.39E-43	down
BMB171_RS07140	324	phosphoribosyl-ATP pyrophosphatase	36.7	15.9	127.7	67.3	-1.95	1.39E-20	down
BMB171_RS07145	1017	histidinol-phosphatase	54.1	45.3	160.7	121.6	-1.53	6.25E-63	down
BMB171_RS07670	1113	histidinol-phosphate aminotransferase	75.4	73.5	219.3	262.6	-1.68	1.12E-131	down
BMB171_RS11820	585	caffeoyl-CoA O-methyltransferase	515.6	483.2	1084.2	1060.8	-1.11	2.83E-163	down
BMB171_RS13920	1485	aldehyde dehydrogenase	20.3	22.7	42.2	48.7	-1.07	1.16E-17	down
BMB171_RS15195	576	cephalosporin hydroxylase	236.7	246.6	14.4	9.6	4.31	4.45E-181	up
**Tyrosine metabolism (btb00350)**									
BMB171_RS01295	1119	4-hydroxyphenylpyruvate dioxygenase	781.1	770.1	2475.9	2366.5	-1.65	0	down
BMB171_RS01305	1173	homogentisate 1,2-dioxygenase	1185.1	1156.6	4488.0	4485.3	-1.94	0	down
BMB171_RS01900	1452	succinate-semialdehyde dehydrogenase	304.7	285.2	942.4	878.6	-1.63	0	down
BMB171_RS06145	753	methyltransferase	34.5	42.0	111.9	99.8	-1.47	2.30E-33	down
BMB171_RS07670	1113	histidinol-phosphate	75.4	73.5	219.3	262.6	-1.68	1.12E-131	down

		aminotransferase							
BMB171_RS07815	1188	aspartate aminotransferase	471.2	468.9	143.3	163.8	1.62	2.18E-260	up
BMB171_RS09960	1464	amine oxidase	8.9	11.7	21.2	28.4	-1.24	2.43E-12	down
BMB171_RS11820	585	caffeoyl-CoA O-methyltransferase	515.6	483.2	1084.2	1060.8	-1.11	2.83E-163	down
BMB171_RS12570	1434	4-hydroxyphenylacetate 3-hydroxylase	226.4	239.8	64.7	58.0	1.92	9.60E-198	up
BMB171_RS15195	576	cephalosporin hydroxylase	236.7	246.6	14.4	9.6	4.31	4.45E-181	up
**Phenylalanine metabolism (btb00360)**									
BMB171_RS01295	1119	4-hydroxyphenylpyruvate dioxygenase	781.1	770.1	2475.9	2366.5	-1.65	0	down
BMB171_RS07670	1113	histidinol-phosphate aminotransferase	75.4	73.5	219.3	262.6	-1.68	1.12E-131	down
BMB171_RS07815	1188	aspartate aminotransferase	471.2	468.9	143.3	163.8	1.62	2.18E-260	up
BMB171_RS09960	1464	amine oxidase	8.9	11.7	21.2	28.4	-1.24	2.43E-12	down
BMB171_RS10955	876	D-amino acid aminotransferase	55.1	45.1	113.0	114.6	-1.19	4.43E-30	down
BMB171_RS21730	1755	phenylalanine 4-monooxygenase	103.1	117.4	366.3	387.5	-1.77	0	down
BMB171_RS26100	921	D-amino acid aminotransferase	25.8	23.1	66.7	59.0	-1.37	2.68E-22	down
BMB171_RS26695	852	3-hydroxybutyryl-CoA dehydrogenase	709.0	702.3	1929.4	1455.1	-1.28	0	down
**Tryptophan metabolism (btb00380)**									
BMB171_RS06495	1368	aldehyde dehydrogenase	2.2	6.8	15.3	17.7	-1.85	9.77E-14	down
BMB171_RS09960	1464	amine oxidase	8.9	11.7	21.2	28.4	-1.24	2.43E-12	down
BMB171_RS13580	630	metal-dependent hydrolase	55.4	44.2	104.6	107.2	-1.09	4.22E-18	down
BMB171_RS13920	1485	aldehyde dehydrogenase	20.3	22.7	42.2	48.7	-1.07	1.16E-17	down
BMB171_RS15870	3198	NADPH-cytochrome P450 reductase	121.3	117.7	373.2	332.2	-1.57	0	down
BMB171_RS26700	1182	acetyl-CoA acetyltransferase	443.8	422.2	1363.3	942.2	-1.43	0	down

**Phenylalnine, tyrosine and tryptophan biosynthesis (00400)**									
BMB171_RS06290	1026	anthranilate phosphoribosyltransferase	0.0	2.0	6.3	4.7	-2.42	1.13E-05	down
BMB171_RS06295	762	indole-3-glycerol-phosphate synthase	2.9	7.2	17.5	9.5	-1.42	3.47E-05	down
BMB171_RS06300	615	N-(5'-phosphoribosyl)anthranilate	0.6	4.5	13.5	13.0	-2.34	2.61E-07	down
BMB171_RS06305	1194	tryptophan synthase subunit beta	11.5	9.8	29.7	27.3	-1.43	2.35E-14	down
BMB171_RS06310	777	tryptophan synthase subunit alpha	11.0	15.0	55.9	48.1	-2.00	2.19E-26	down
BMB171_RS07660	1173	chorismate synthase	8.6	9.7	22.9	18.0	-1.18	4.11E-08	down
BMB171_RS07670	1113	histidinol-phosphate aminotransferase	75.4	73.5	219.3	262.6	-1.68	1.12E-131	down
BMB171_RS07815	1188	aspartate aminotransferase	471.2	468.9	143.3	163.8	1.62	2.18E-260	up
BMB171_RS09960	1464	amine oxidase	8.9	11.7	21.2	28.4	-1.24	2.43E-12	down
BMB171_RS21730	1755	phenylalanine 4-monooxygenase	103.1	117.4	366.3	387.5	-1.77	0	down
**Xenobiotics biodegradation and metabolism**									
**Benzoate degradation (btb00362)**									
BMB171_RS01960	1110	mandelate racemase/muconate lactonizing family protein	7.7	12.4	22.1	18.7	-1.02	2.50E-06	down
BMB171_RS26695	852	3-hydroxybutyryl-CoA dehydrogenase	709.0	702.3	1929.4	1455.1	-1.28	0	down
BMB171_RS26700	1182	acetyl-CoA acetyltransferase	443.8	422.2	1363.3	942.2	-1.43	0	down
**Aminobenzoate degradation (btb00627)**									
BMB171_RS11140	699	3-keto-6-acetamidohexanoate cleavage protein	91.4	110.7	286.7	260.6	-1.44	7.64E-76	down
BMB171_RS13040	1215	cytochrome P450	2.4	3.4	11.7	13.1	-2.07	6.63E-11	down
BMB171_RS15870	3198	NADPH-cytochrome P450 reductase	121.3	117.7	373.2	332.2	-1.57	0	down
BMB171_RS21680	1386	alkaline phosphatase	13.7	9.2	30.7	30.2	-1.43	1.79E-17	down

**Fluorobenzoate degradation (btb00364)**									
BMB171_RS01960	1110	mandelate racemase/muconate lactonizing family protein	7.7	12.4	22.1	18.7	-1.02	2.50E-06	down
**Chloroalkane and chloroalkene degradation (btb00625)**									
BMB171_RS06495	1368	aldehyde dehydrogenase	2.2	6.8	15.3	17.7	-1.85	9.77E-14	down
BMB171_RS13920	1485	aldehyde dehydrogenase	20.3	22.7	42.2	48.7	-1.07	1.16E-17	down
BMB171_RS18435	1164	NADH-dependent butanol dehydrogenase A	25.2	29.2	70.8	62.2	-1.29	6.29E-27	down
**Chlorocyclohexane and chlorobenzene degradation (btb00361)**									
BMB171_RS01960	1110	mandelate racemase/muconate lactonizing family protein	7.7	12.4	22.1	18.7	-1.02	2.50E-06	down
**Toluene degradation (btb00623)**									
BMB171_RS01960	1110	mandelate racemase/muconate lactonizing family protein	7.7	12.4	22.1	18.7	-1.02	2.50E-06	down
**Styrene degradation (btb00643)**									
BMB171_RS01305	1173	homogentisate 1,2-dioxygenase	1185.1	1156.6	4488.0	4485.3	-1.94	0	down
**Bisphenol degradation (btb00363)**									
BMB171_RS13040	1215	cytochrome P450	2.4	3.4	11.7	13.1	-2.07	6.63E-11	down
BMB171_RS18435	1164	NADH-dependent butanol dehydrogenase A	25.2	29.2	70.8	62.2	-1.29	6.29E-27	down
**Polycyclic aromatic hydrocarbon degradation (btb00624)**									
BMB171_RS06145	753	methyltransferase	34.5	42.0	111.9	99.8	-1.47	2.30E-33	down
BMB171_RS11820	585	caffeoyl-CoA O-methyltransferase	515.6	483.2	1084.2	1060.8	-1.11	2.83E-163	down
BMB171_RS13040	1215	cytochrome P450	2.4	3.4	11.7	13.1	-2.07	6.63E-11	down
BMB171_RS15195	576	cephalosporin hydroxylase	236.7	246.6	14.4	9.6	4.31	4.45E-181	up
**Metabolism of xenobiotics by cytochrome P450 (btb00980)**									

BMB171_RS13760	1194	macrolide glycosyltransferase	0.6	1.7	4.7	4.1	-1.88	0.00037797	down
**Drug metabolism-cytochrome P450 (btb00982)**									
BMB171_RS13760	1194	macrolide glycosyltransferase	0.6	1.7	4.7	4.1	-1.88	0.00037797	down
**Drug metabolism – other enzymes (btb00983)**									
BMB171_RS21450	399	cytidine deaminase	61.4	54.3	120.0	117.2	-1.04	2.88E-12	down
BMB171_RS21845	639	uridine kinase	42.4	37.7	83.7	80.7	-1.04	1.45E-13	down
BMB171_RS24005	528	hypoxanthine-guanine phosphoribosyltransferase	9.8	13.0	30.2	38.7	-1.57	3.57E-09	down
BMB171_RS07765	1203	tRNA CCA-pyrophosphorylase	27.8	26.6	86.7	80.3	-1.62	3.88E-48	down
BMB171_RS20575	924	ribonuclease Z	3.2	3.3	9.3	10.9	-1.61	2.10E-05	down
**Biosynthesis of other secondary metabolism**									
**Stilbenoid, diarylheptanoid and gingerol biosynthesis (00945)**									
BMB171_RS13040	1215	cytochrome P450	2.4	3.4	11.7	13.1	-2.07	6.63E-11	down
**Isoquinoline alkaloid biosynthesis (btb00950)**									
BMB171_RS07815	1188	aspartate aminotransferase	471.2	468.9	143.3	163.8	1.62	2.18E-260	up
BMB171_RS09960	1464	amine oxidase	8.9	11.7	21.2	28.4	-1.24	2.43E-12	down
**Tropane, piperidine and pyridine alkaloid biosynthesis (btb00960)**									
BMB171_RS07815	1188	aspartate aminotransferase	471.2	468.9	143.3	163.8	1.62	2.18E-260	up
BMB171_RS07670	1113	histidinol-phosphate aminotransferase	75.4	73.5	219.3	262.6	-1.68	1.12E-131	down
**Penicillin and cephalosporin biosynthesis (btb00311)**									
BMB171_RS19285	915	aspartate carbamoyltransferase	22.3	18.0	60.4	46.9	-1.43	2.25E-20	down
**Glycine, serine and threonine metabolism (btb00260)**									
BMB171_RS16015	2391	penicillin acylase II	16.6	15.8	90.1	55.6	-2.20	6.00E-125	down
**Streptomycin biosynthesis (btb00521)**									
BMB171_RS06180	738	glucose-1-phosphate	222.5	184.1	1037.3	755.0	-2.16	0	down

		thymidylyltransferase							
BMB171_RS06190	969	dTDP-glucose 4,6-dehydratase	356.1	303.1	1205.1	1087.1	-1.81	0	down
BMB171_RS06195	855	dTDP-4-dehydrorhamnose reductase	292.4	275.5	954.4	870.0	-1.69	0	down
BMB171_RS26275	855	dTDP-4-dehydrorhamnose 3,5-epimerase	28.7	19.3	43.2	53.1	-1.00	3.39E-10	down
BMB171_RS26280	561	glucose-1-phosphate thymidylyltransferase	9.9	14.7	29.0	22.8	-1.07	9.01E-05	down
**Novobiocin biosynthesis (btb00524)**									
BMB171_RS07815	1188	aspartate aminotransferase	471.2	468.9	143.3	163.8	1.62	2.18E-260	up
BMB171_RS07670	1113	histidinol-phosphate aminotransferase	75.4	73.5	219.3	262.6	-1.68	1.12E-131	down
**Transcription**									
**RNA polymerase (btb03020)**									
BMB171_RS00615	3255	DNA-directed RNA polymerase subunit beta'	587.4	597.3	1163.5	1299.3	-1.05	0	down
BMB171_RS00785	945	DNA-directed RNA polymerase subunit alpha	1414.0	1355.5	3343.1	2968.3	-1.20	0	down
BMB171_RS19185	213	DNA-directed RNA polymerase subunit omega	29.6	21.0	65.2	53.7	-1.25	3.58E-05	down
BMB171_RS26675	489	DNA-directed RNA polymerase subunit delta	191.4	203.2	435.8	432.7	-1.14	1.57E-58	down
**Lipid metabolism**									
**Fatty acid biosynthesis (btb00061)**									
BMB171_RS05975	933	3-oxoacyl-(acyl carrier protein) synthase III	88.0	89.6	297.8	242.4	-1.62	1.53E-118	down
BMB171_RS06200	771	enoyl-(acyl carrier protein) reductase	229.8	225.7	484.9	434.3	-1.02	1.05E-81	down

BMB171_RS09125	759	short chain dehydrogenase	28.9	30.8	54.5	66.2	-1.00	3.35E-11	down
BMB171_RS12335	1338	acetyl-CoA carboxylase biotin carboxylase subunit	490.3	514.3	1274.6	1244.6	-1.33	0	down
BMB171_RS18760	714	3-ketoacyl-(acyl-carrier-protein) reductase	9.4	7.2	27.3	20.4	-1.55	1.16E-08	down
BMB171_RS19085	741	3-ketoacyl-(acyl-carrier-protein) reductase	150.4	145.7	368.2	326.2	-1.24	5.14E-81	down
BMB171_RS19090	945	acyl-carrier-protein S-malonyltransferase	125.0	120.8	300.2	271.2	-1.22	1.83E-83	down
BMB171_RS23010	759	3-ketoacyl-(acyl-carrier-protein) reductase	1185.9	1284.9	417.8	380.5	1.63	0	up
**Fatty acid degradation (btb00071)**									
BMB171_RS06495	1368	aldehyde dehydrogenase	2.2	6.8	15.3	17.7	-1.85	9.77E-14	down
BMB171_RS11350	1710	acyl-CoA dehydrogenase	194.6	202.9	517.1	476.5	-1.33	5.45E-294	down
BMB171_RS13920	1485	aldehyde dehydrogenase	20.3	22.7	42.2	48.7	-1.07	1.16E-17	down
BMB171_RS15870	3198	NADPH-cytochrome P450 reductase	121.3	117.7	373.2	332.2	-1.57	0	down
BMB171_RS22500	777	enoyl-CoA hydratase	853.4	848.4	1824.3	1763.1	-1.08	0	down
BMB171_RS22510	1749	acyl-CoA synthase	241.5	222.2	1086.4	1058.1	-2.21	0	down
BMB171_RS26700	1182	acetyl-CoA acetyltransferase	443.8	422.2	1363.3	942.2	-1.43	0	down
**Synthesis and degradation of ketone bodies (btb00072)**									
BMB171_RS20010	789	3-hydroxybutyrate dehydrogenase	22.6	16.6	57.3	65.8	-1.65	2.70E-24	down
BMB171_RS26700	1182	acetyl-CoA acetyltransferase	443.8	422.2	1363.3	942.2	-1.43	0	down
**Steroid hormone biosynthesis (btb00140)**									
BMB171_RS13760	1194	macrolide glycosyltransferase	0.6	1.7	4.7	4.1	-1.88	0.00037797	down
**Glycerolipid metabolim (btb00561)**									
BMB171_RS02575	1167	diacylglycerol glucosyltransferase	28.0	18.0	53.7	61.7	-1.33	2.05E-24	down

BMB171_RS05010	999	dihydroxyacetone kinase	5.9	2.4	10.4	11.4	-1.40	3.25E-05	down
BMB171_RS05280	1491	glycerol kinase	80.2	75.4	173.4	149.8	-1.06	4.93E-60	down
BMB171_RS06495	1368	aldehyde dehydrogenase	2.2	6.8	15.3	17.7	-1.85	9.77E-14	down
BMB171_RS12685	1242	lipase	3.0	3.0	11.2	5.6	-1.51	1.22E-05	down
BMB171_RS13920	1485	aldehyde dehydrogenase	20.3	22.7	42.2	48.7	-1.07	1.16E-17	down
BMB171_RS17855	597	glycerol-3-phosphate acyltransferase PlsY	7.5	8.1	13.9	24.4	-1.27	0.000100176	down
BMB171_RS19095	993	glycerol-3-phosphate acyltransferase PlsX	88.3	80.7	218.5	185.2	-1.27	6.01E-66	down
**Glycerophospholipid metabolism (btb00564)**									
BMB171_RS04995	534	CDP-diacylglycerol-serine O-phosphatidyltransferase	9.0	13.5	32.1	20.8	-1.24	1.28E-05	down
BMB171_RS06070	1545	cardiolipin synthetase	62.3	67.7	17.6	13.9	2.03	7.64E-65	up
BMB171_RS07160	702	CDP-diacylglycerol--serine O-phosphatidyltransferase	82.0	75.0	150.3	171.2	-1.03	5.16E-27	down
BMB171_RS07605	1023	NAD(P)H-dependent glycerol-3-phosphate dehydrogenase	555.2	601.4	1233.7	1320.9	-1.14	0	down
BMB171_RS17855	597	glycerol-3-phosphate acyltransferase PlsY	7.5	8.1	13.9	24.4	-1.27	0.000100176	down
BMB171_RS18940	792	phosphatidate cytidylyltransferase	24.9	18.7	42.9	51.2	-1.11	8.33E-11	down
BMB171_RS19095	993	glycerol-3-phosphate acyltransferase PlsX	88.3	80.7	218.5	185.2	-1.27	6.01E-66	down
BMB171_RS23675	711	lysophospholipase L2	11.0	16.9	63.2	78.9	-2.32	1.19E-38	down
BMB171_RS26710	1194	cardiolipin synthetase	20.2	15.3	49.3	55.7	-1.57	1.00E-28	down
**Linoleic acid metabolism (btb00591)**									
BMB171_RS18435	1164	NADH-dependent butanol	25.2	29.2	70.8	62.2	-1.29	6.29E-27	down

		dehydrogenase A							
**Biosynthesis of unsaturated fatty acids (btb01040)**									
BMB171_RS09125	759	short chain dehydrogenase	28.9	30.8	54.5	66.2	-1.00	3.35E-11	down
BMB171_RS18760	714	3-ketoacyl-(acyl-carrier-protein) reductase	9.4	7.2	27.3	20.4	-1.55	1.16E-08	down
BMB171_RS19085	741	3-ketoacyl-(acyl-carrier-protein) reductase	150.4	145.7	368.2	326.2	-1.24	5.14E-81	down
BMB171_RS23010	759	3-ketoacyl-(acyl-carrier-protein) reductase	1185.9	1284.9	417.8	380.5	1.63	0	up
**Cell growth and death**									
**Cell cycle-Caulobacter (04112)**									
BMB171_RS00005	1341	chromosomal replication initiation protein	6.1	3.6	12.6	9.6	-1.22	1.05E-05	down
BMB171_RS13730	582	ATP-dependent Clp protease proteolytic subunit	427.7	449.7	67.5	98.8	2.42	1.13E-201	up
BMB171_RS19375	1308	cell division protein FtsA	319.8	306.8	703.6	788.2	-1.25	1.73E-304	down
BMB171_RS19380	771	cell division protein FtsQ	11.1	11.6	33.0	31.4	-1.51	1.38E-11	down
BMB171_RS19390	1158	N-acetylglucosaminyl transferase	5.1	6.8	16.3	16.1	-1.43	1.76E-08	down
BMB171_RS19395	1092	stage V sporulation protein E	92.9	81.9	162.1	196.0	-1.03	1.10E-45	down
BMB171_RS19580	1179	cell division protein FtsW	21.4	23.0	62.4	72.0	-1.59	1.26E-36	down
BMB171_RS22285	2199	ATP-dependent protease La	40.7	42.7	105.1	95.3	-1.27	3.02E-72	down
BMB171_RS22295	1260	ATP-dependent protease ATP-binding protein	326.4	349.3	827.3	879.5	-1.33	0	down
